# Quadratic Fermi node in a 3D strongly correlated semimetal

**DOI:** 10.1038/ncomms10042

**Published:** 2015-12-07

**Authors:** Takeshi Kondo, M. Nakayama, R. Chen, J. J. Ishikawa, E.-G. Moon, T. Yamamoto, Y. Ota, W. Malaeb, H. Kanai, Y. Nakashima, Y. Ishida, R. Yoshida, H. Yamamoto, M. Matsunami, S. Kimura, N. Inami, K. Ono, H. Kumigashira, S. Nakatsuji, L. Balents, S. Shin

**Affiliations:** 1 ISSP, University of Tokyo, Kashiwa, Chiba 277-8581, Japan; 2 Physics Department, University of California, Santa Barbara, California 93106, USA; 3 Physics Department, University of California, Berkeley, California 94720, USA; 4 Molecular Foundry, Lawrence Berkeley National Laboratory, Berkeley, California 94720, USA; 5 Department of Physics, Korea Advanced Institute of Science and Technology, Daejeon 305-701, Korea; 6 Physics Department, Faculty of Science, Beirut Arab University, P. O. Box 11-5020 Beirut, Lebanon; 7 UVSOR Facility, Institute for Molecular Science, Okazaki 444-8585, Japan; 8 Energy Materials Laboratory, Toyota Technological Institute, Nagoya 468-8511, Japan; 9 Graduate School of Frontier Biosciences, Osaka University, Suita, Osaka 565-0871, Japan; 10 Institute of Materials Structure Science, High Energy Accelerator Research Organization (KEK), Tsukuba, Ibaraki 305-0801, Japan; 11 PRESTO, Japan Science and Technology Agency (JST), 4-1-8 Honcho Kawaguchi, Saitama 332-0012, Japan; 12 Kavli Institute for Theoretical Physics, Santa Barbara, California 93106, USA

## Abstract

Strong spin–orbit coupling fosters exotic electronic states such as topological insulators and superconductors, but the combination of strong spin–orbit and strong electron–electron interactions is just beginning to be understood. Central to this emerging area are the 5*d* transition metal iridium oxides. Here, in the pyrochlore iridate Pr_2_Ir_2_O_7_, we identify a non-trivial state with a single-point Fermi node protected by cubic and time-reversal symmetries, using a combination of angle-resolved photoemission spectroscopy and first-principles calculations. Owing to its quadratic dispersion, the unique coincidence of four degenerate states at the Fermi energy, and strong Coulomb interactions, non-Fermi liquid behaviour is predicted, for which we observe some evidence. Our discovery implies that Pr_2_Ir_2_O_7_ is a parent state that can be manipulated to produce other strongly correlated topological phases, such as topological Mott insulator, Weyl semimetal, and quantum spin and anomalous Hall states.

## Abstract (web summary)

5d transition metal iridates provide a platform to study the combined effects of strong spin orbit coupling and strong electronic correlations. Here, the authors find a quadratic band touching in the band structure of Pr_2_Ir_2_O_7_, suggesting it may be tuned to form various strongly correlated topological phases.

## Graphical abstract

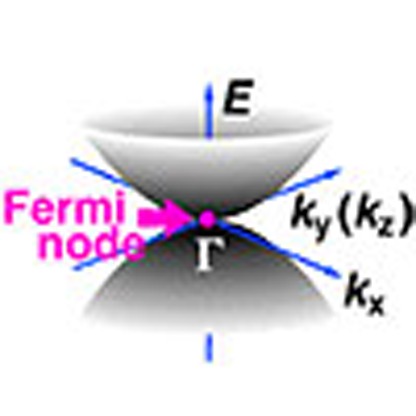

Following the discovery of topological insulators^1^,^2^,^3^,^4^, the next frontier is the regime in which both spin–orbit coupling and correlation effects are strong^5^,^6^,^7^,^9^,^10^,^11^,^12^. Theory has suggested that the pyrochlore iridates, a family of cubic 5*d* transition metal oxides^13^,^14^, may realize both band inversion, the essential ingredient of topological insulators and strong correlations^9^,^12^. Empirical evidence for the latter is plentiful. Notably, Pr_2_Ir_2_O_7_ appears to be proximate to an interaction-driven antiferromagnetic quantum critical point tuned by the A-site ionic radius, which is located between the two ions with largest radii, A=Pr and A=Nd^14^,^15^. It is the only compound among the series in which the iridium electrons remain paramagnetic and itinerant down to the lowest measured temperatures. It displays bad metallic behaviour and non-trivial spontaneous Hall transport, suggesting strong correlations^6^,^15^,^16^. Moreover, recent thermodynamic measurements have revealed zero-field quantum criticality without tuning^17^.

The phenomenological suggestion of Moon *et al.*^11^, whose implications are summarized in Fig. 1, is that the Fermi surface of Pr_2_Ir_2_O_7_ contains a single Fermi node at the Γ point, which emerges as the touching point of two quadratically dispersing conduction and valence bands^18^. The presence of this touching is actually required by symmetry and group theory (the quadruplet at the zone centre lies in the Γ_8_ representation of the double group of O_h_), but its location directly at the Fermi energy was an *ad hoc* theoretical assumption. If the assumption is correct, Pr_2_Ir_2_O_7_ becomes a strongly correlated analogue of HgTe^19^,^20^, which has a mathematically identical quadratic node at the Fermi energy, and implies that Pr_2_Ir_2_O_7_ should be tunable into various topological states (see Fig. 1). Furthermore, theory has predicted that the quadratic nodal semimetal itself is fundamentally altered by long-range Coulomb interactions (negligible in HgTe due to the large dielectric constant, but not so here), becoming a non-Fermi liquid state. Thus the Fermi node, if correct, means that Pr_2_Ir_2_O_7_ is a natural parent material for strongly interacting topological phases and non-Fermi liquid states.

In this paper, we focus on the phenomena of band inversion, and present theoretical calculations and experimental angle-resolved photoemission spectroscopy (ARPES) spectra, which support it in the form of electronic structure with a node at the Fermi energy. We also observe strongly temperature-dependent single-particle spectral weight and lineshape structure in ARPES, which suggest electronic correlations and coupling to collective modes.

## Results

### Fermi node state expected by band calculations

**Figure 2:**
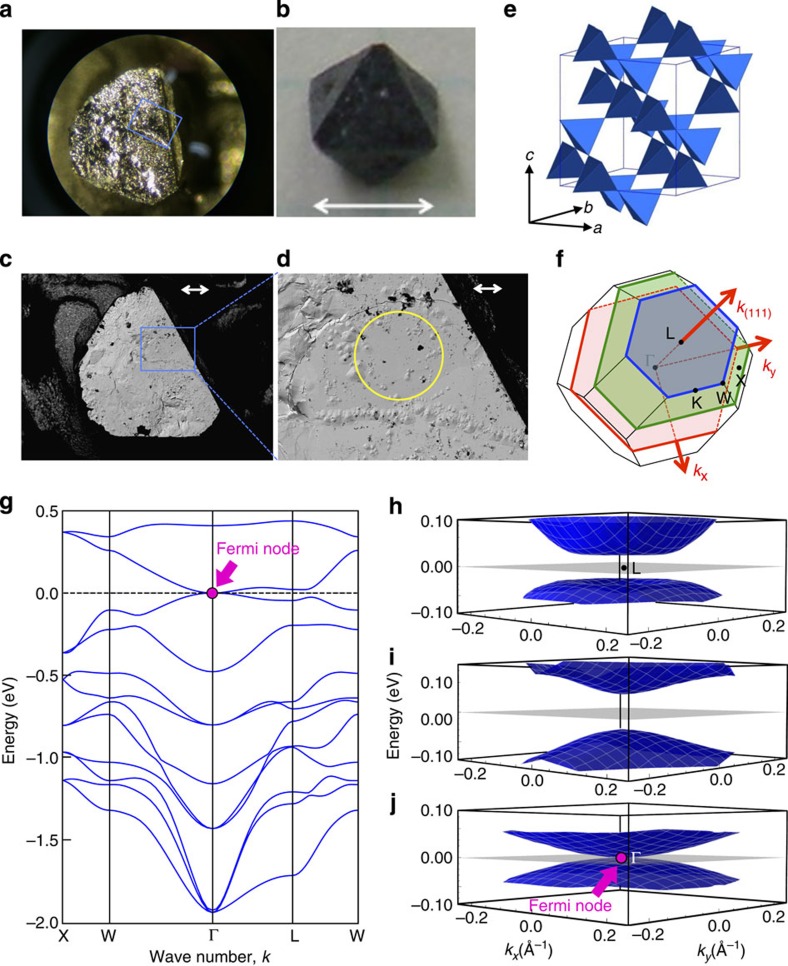
Single-crystal and first-principles band calculation for Pr_2_Ir_2_O_7_. (**a**,**b**) Photos of the cleaved (111) surface measured by ARPES and single crystal we used, respectively. The top surface with a triangle shape is the (111) plane. (**c**) The SEM image for the cleaved surface of **a**. (**d**) The same image as **c**, but magnified in a small region marked by a blue square in **a** and **c**. The arrows in **b**,**c** and **d** indicate dimensions of 1 mm, 200 and 50 μm, respectively. (**e**) Crystal structure of Pr_2_Ir_2_O_7_ showing the pyrochlore lattice of Ir (or Pr) atoms. (**f**) Brillouin zone. (**g**) Band dispersion along high-symmetry lines obtained by the first-principles calculation. Band dispersions in three *k*_*x*_–*k*_*y*_ planes (coloured in **f**) perpendicular to the *k*_(111)_ direction: crossing L (**h**), crossing Γ (**j**) and crossing between these two points (**i**). The Fermi node is indicated with a magenta circle and arrow in **g** and **j**.

In support of this proposition, we first present *ab initio* electronic structure calculations in the paramagnetic state. As detailed in the Supplementary Fig. 1, we show that the quadratic band touching systematically approaches the Fermi level with increasing A-site ionic radius, reaching it for *A*=Pr. The corresponding band dispersion is shown in Fig. 2g along the high-symmetry lines. The nodal Fermi point and quadratic band touching at Γ is clearly visible at the Fermi energy. The bandwidth is narrower in energy than that of HgTe^21^ by one order of magnitude, reflecting the localized nature of the 5*d* orbitals in Pr_2_Ir_2_O_7_. Theoretical uncertainty remains, however, as discussed in the Supplementary Note 1 (also see Supplementary Figs 1 and 2), so direct experimental evidence for the unique Fermi node state is strongly desired.

ARPES is a powerful technique to directly observe the electronic structure of matter^22^,^23^. One incident photon energy corresponds to one *k*_*z*_ value in solid, thus the momentum space observed at a fixed photon energy is limited to a *k*_*x*_–*k*_*y*_ plane at a fixed *k*_*z*_. To locate the Γ-point, therefore, sweeping the photon energy is required. Figure 2a,b shows the photos of the cleavage surface with the (111) plane measured by ARPES and the high-quality single crystal we used, respectively. While the cleaved surface looks rough in the photo, the scanning electron microscope image of it (Fig. 2c,d) exhibits very flat parts in the multiple locations, which are large enough compared with the photon beam size (∼150 μm, marked with a yellow circle in Fig. 2d). Several *k*_*x*_–*k*_*y*_ sheets perpendicular to *k*_(111)_ measured at different photon energies are coloured in Fig. 2f. The band dispersions obtained by the *ab initio* calculation for these sheets are plotted in Fig. 2h–j. The experimentally obtained energy dispersion is expected to have a large gap when the *k*_(111)_ location is far from the Γ. As *k*_(111)_ is reduced, the gap decreases and eventually vanishes at Γ, where the two parabolic dispersions touch at *E*_F_ (Fig. 2j).

### Observation of Fermi node in Pr_2_Ir_2_O_7_ by ARPES

**Figure 3:**
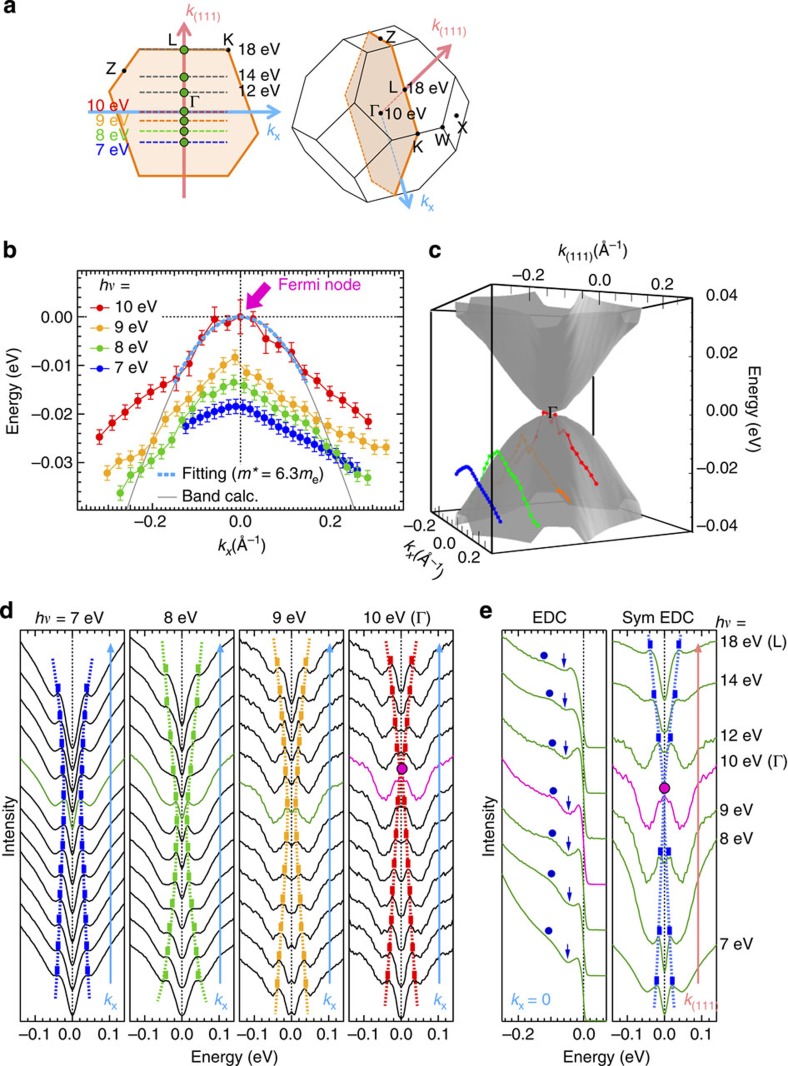
ARPES spectra revealing a quadratic Fermi node in the 3D band of Pr_2_Ir_2_O_7_. (**a**) Brillouin zone, showing a momentum sheet along which ARPES data were measured. The momentum cuts at *hν*=10 and 18 eV crosses the Γ and L points in the 1st Brillouin zone. (**b**) Energy dispersions along the *k*_*x*_ direction measured at *hν*=7, 8, 9 and 10 eV. The corresponding momentum cuts are indicated in **a** by dashed coloured lines. The band dispersion obtained by the first-principles band calculation is superimposed (grey curve). The data close to *E*_F_ is fitted by a parabolic function, *ɛ*(*k*)∝*k*^2^ (light-blue dotted curve). The estimated effective mass at Γ, *m*_*eff*_=6.3*m*_0_ (*m*_0_: free electron mass), is in agreement with the band calculation. (**c**) The calculated band dispersion in the *k*_*x*_−*k*_(111)_ sheet, painted with orange in **a**. On it, the ARPES data in **b** are plotted. (**d**) The ARPES data (symmetrized energy distribution curves (EDCs)) along the *k*_*x*_ direction measured at several photon energies. All the spectra shown were accumulated at *T*=15 K. The energy positions of spectral peaks are marked by bars and dashed curves. (**e**) EDCs and the corresponding symmetrized EDCs along the Γ–L direction, measured at *k* points marked with green circles in **a**. The energy positions of dip and hump, suggesting mode coupling, are marked by arrows and circles, respectively, on EDCs. Error bars in **b** represent uncertainty in estimating the spectral peak positions.

To observe the Fermi node, we measured the ARPES spectra at various photon energies: *hν*=7 eV (a laser source) and 21.2 eV (a He lamp) in the lab system, and *hv*=8∼18 eV and 39∼60 eV from two different synchrotron facilities. The results for the 1st Brillouin zone (BZ) are shown in Fig. 3d, where the energy distribution curves (EDCs) along *k*_*x*_ (defined in Fig. 3a) are plotted. The observed momentum cut shifts with photon energy along the *k*_(111)_ axis within a momentum sheet crossing Γ, L and K points (coloured dashed lines in Fig. 3a). The spectra are symmetrized to remove the effect of the Fermi cutoff: the EDC is flipped about *E*_F_ and added to the original one. This method is widely accepted as a means of determining the presence of a gap (or a gap node). Small but clear quasiparticle peaks are seen for all the spectra, which allows us to determine the energy dispersion, as marked by bars and dotted curves. At *hν*=7 eV (see Fig. 3d), a large gap of ∼20 meV is opened at *k*_*x*_=0 (green curve), and a parabolic dispersion is obtained. The most significant finding is that the parabolic dispersion moves towards the *E*_F_ with increasing incident photon energy (or increasing *k*_(111)_), and it eventually touches *E*_F_ at *hν*=10 eV. This behaviour is more clearly demonstrated in Fig. 3b,c, where the band dispersion determined from the peak energies of spectra at 7, 8, 9 and 10 eV are plotted. As the photon energy is further increased, the dispersion moves away from *E*_F_ again, following the quadratic dispersion along *k*_(111)_ as shown in Fig. 3e (also see Supplementary Figs 3 and 4; Supplementary Note 2). We have also examined the dispersion along a different momentum sheet crossing Γ, L and W points (see Supplementary Fig. 5 and Supplementary Note 2) for a different piece of sample. This time the photon energy was swept by a finer step (≤0.5 eV), and the Fermi node was again detected at *hν*=10.5 eV. While it is not possible to eliminate a slight uncertainty in the exact photon energy, which yields *k*_(111)_=0, owing to the broad shape of the spectral peak, our data show that the three-dimensional band structure of Pr_2_Ir_2_O_7_ has the theoretically predicted Fermi point at the momentum reached by *hv*∼10 eV, which is thus assigned to be Γ. Other scans of different *k*_(111)_ values up to the L point in the 1st BZ, which is reached at *hν*=18 eV, revealed no other states touching or crossing *E*_F_ (see Supplementary Fig. 3). This absence of other bands crossing *E*_F_ is another consistency condition on the Fermi node model, which requires this situation by state counting and charge neutrality.

**Figure 4:**
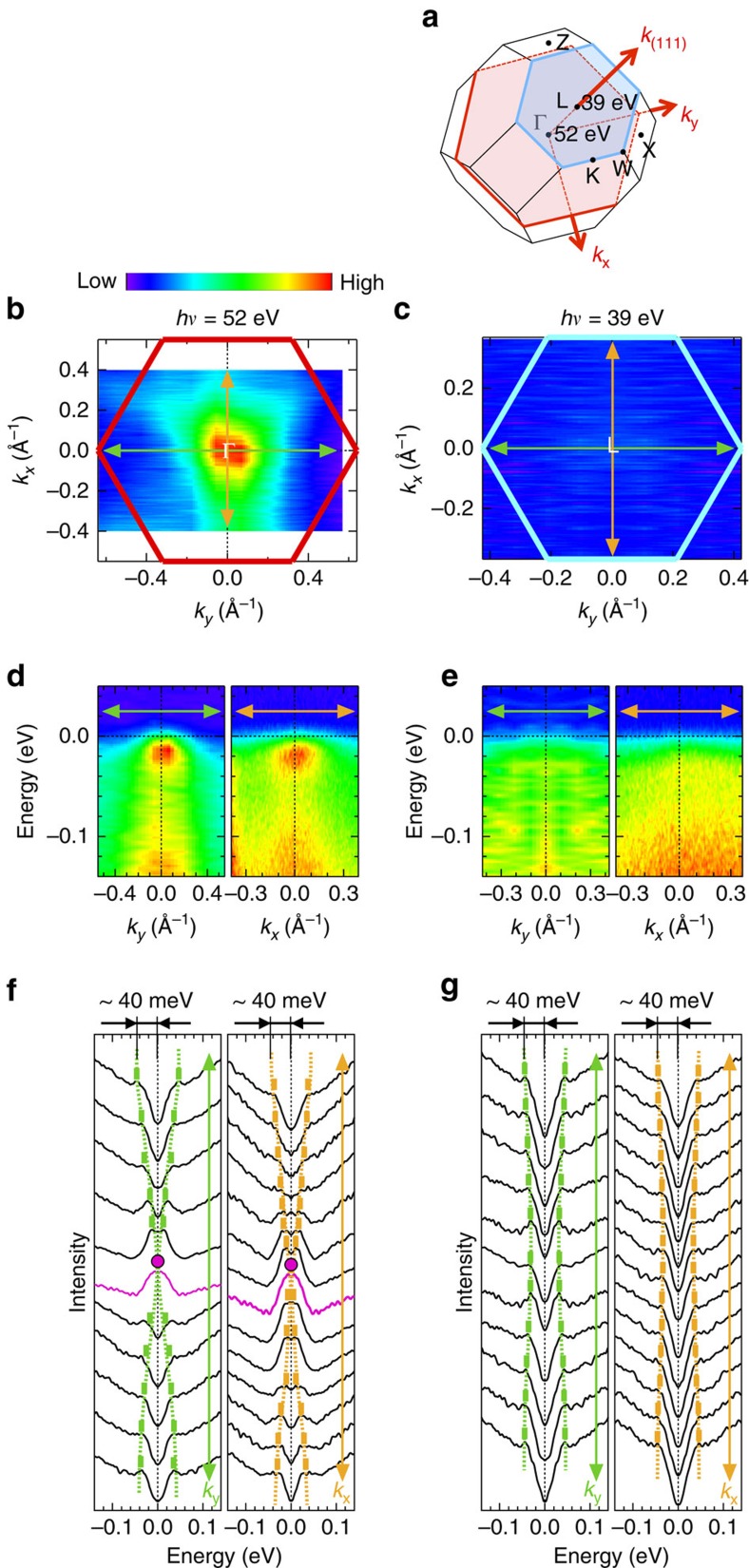
ARPES intensity mapping at the Fermi level across Γ and L points. (**a**) Brillouin zone, showing momentum sheets along which ARPES data were measured. The momentum cuts at *hν*=52 and 39 eV crosses the Γ and L points, respectively, in the 3rd Brillouin zone. (**b**,**c**) ARPES intensities at *E*_F_ in the *k*_*x*_−*k*_*y*_ sheet crossing Γ and L measured at *hν*=52 and 39 eV, respectively. For the data at *hν*=39 eV, only the positive side of *k*_*y*_ were measured, and it is reflected to the negative side. (**d**) ARPES dispersion maps for *hν*=52 eV, measured along *k*_*y*_ and *k*_*x*_ crossing Γ (green and orange arrows in **b**, respectively). (**e**) The same data as **d**, but for *hν*=39 eV, which cross the L point. (**f**,**g**) EDCs of **d** and **e**, respectively, symmetrized about *E*_F_. The dimension arrows estimate the bandwidth of ∼40 meV in the occupied side.

Here we validate our conclusion by making several further checks. First, we investigate another BZ to verify the required repetition of the Fermi nodal state along *k*_(111)_. We used higher photon energies, corresponding to the 3rd BZ, and found the Fermi node at the expected Γ point (*hν*=52 eV) (see Supplementary Fig. 6). In the *k*_*x*_–*k*_*y*_ sheet at *hν*=52 eV (Fig. 4a,b), the ARPES intensities at *E*_F_ becomes strongest at the zone centre, and the band touching at *E*_F_ is confirmed in the dispersion maps along *k*_*x*_ and *k*_*y*_ (Fig. 3d) and the corresponding symmetrized EDCs (Fig. 4f, also see the raw EDCs in Supplementary Fig. 7). In contrast, these features are missing in the *k*_*x*_–*k*_*y*_ sheet across L point (*hν*=39 eV) (see Fig. 4c), where a rather flat, gapped dispersion is observed (Fig. 4e,g).

**Figure 5:**
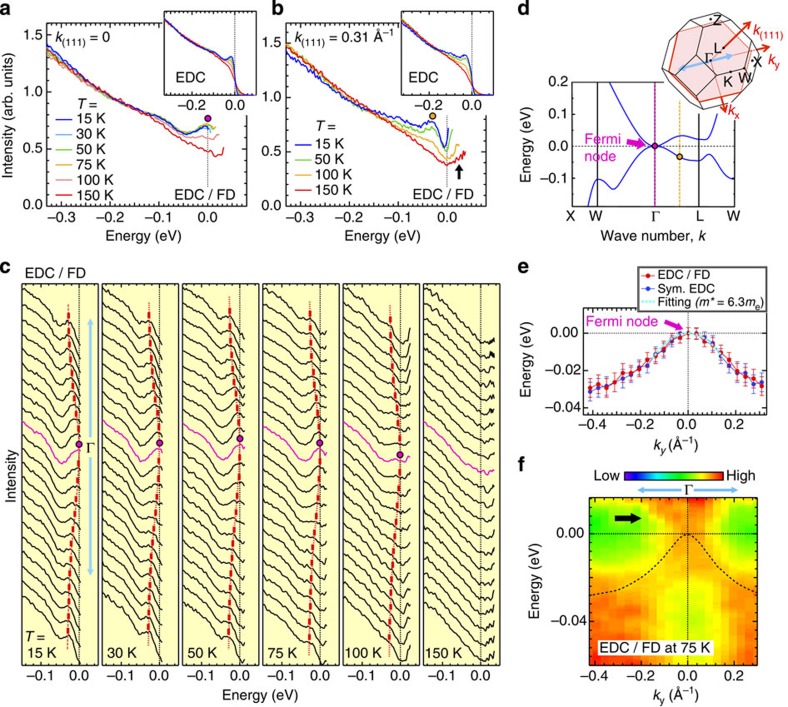
Temperature evolution of ARPES spectra and evidence for the Fermi nodal state. (**a**,**b**) Temperature variation of FD-divided EDCs measured at Γ (*hν*=10.5 eV) and off Γ (*hν*=21.2 eV), respectively. In **d**, the observed *k* points for **a** and **b** are marked by purple and orange dashed lines, respectively. The unoccupied side is displayed up to the energy of 3*k*_*B*_*T*. The spectral peak positions are marked by coloured circles. The original EDCs used for the analysis are plotted in the insets. (**c**) The FD-divided EDCs along a momentum cut indicated in **d** with a light-blue arrow measured at various temperatures. The spectral peak positions are indicated by red bars and dashed curves. The Fermi node is marked by a magenta circle. At *T*=150 K, the spectral peaks are strongly suppressed, and thus the dispersion cannot be determined. (**d**) Band dispersions obtained by the first-principles calculation, magnified in the region close to *E*_F_. In the inset, the Brillouin zone of Pr_2_Ir_2_O_7_ is shown. (**e**) The energy dispersion along a momentum cut crossing Γ (light-blue arrow in **d**), determined from the peak positions of the FD-divided spectra at *T*=75 K in **c** (red circles). The same plots, but determined from the peak positions of symmetrized spectra, are superimposed (blue circles). The quadratic dispersion (light-blue dashed curve) nicely fits the data. (**f**) ARPES dispersion map crossing Γ (light-blue arrow in **d**) measured at *hν*=10.5 eV. The original ARPES intensities are divided by the energy-resolution convoluted Fermi function at the measured temperature of *T*=75 K to remove the cutoff effect near *E*_F_. The thick black arrow points to the remarkable spectral weight above *E*_F_, consistent with the existence of conduction touching band in the unoccupied side. Error bars in **e** represent uncertainty in estimating the spectral peak positions.

Second, we demonstrate that our conclusion is insensitive to the different analytic schemes. This is significant especially because the symmetrization technique is relevant for the particle–hole symmetric state, which is unknown in Pr_2_Ir_2_O_7_. Accordingly, we have tested another widely used measure of dividing the ARPES spectra by the Fermi-Dirac function (FD). Figure 5c plots the EDCs along a momentum cut crossing Γ (light-blue arrow in the inset of Fig. 5d) measured at various temperatures. Instead of symmetrization, the curves are divided by the energy-resolution convoluted Fermi function at the measurement temperatures to remove the effect of the Fermi cutoff. It is clearly seen that the quadratic dispersion touches *E*_F_, in agreement with the earlier analysis. In Fig. 5e, we compare the dispersions determined by the symmetrization and the FD-division methods, and confirm the consistency between the two (see Supplementary Fig. 8 and Supplementary Note 3 for more details).

### Comparison between ARPES results and band calculations

In Fig. 3b, we compare our experimental results near Γ with the *ab initio* dispersion (grey curve), which has a purely quadratic shape of the electronic structure given by *ɛ*(*k*)∝*k*^2^. Close to *E*_F_, we find an agreement between the two, demonstrating that the quadratic curve (light-blue dotted line) fits well to our data with almost the same effective mass, *m*_eff_=6.3*m*_0_ (*m*_0_ is the mass of a free electron). On the other hand, for energy below −0.012 eV, the measured dispersion deviates remarkably from the parabolic shape. This contrasts to the calculation, which matches with *ɛ*(*k*)∝*k*^2^ up to much higher binding energies. It is possible that the deviation is due to correlation effects beyond the band calculation. Intriguingly, the total bandwidth in the occupied side is estimated to be ∼40 meV (arrows in Fig. 4f,g), which is actually much narrower than that (>100 meV) of the band calculation (see Fig. 2g and Supplementary Fig. 9e,h). Band narrowing relative to the density functional theory is indeed a well-known characteristic of correlated electrons. If that is the case, however, the agreement of the effective mass around Γ between the data and calculation would be a coincidence. This is understandable, considering that the band shape of Pr_2_Ir_2_O_7_ with comparable energy scales between the spin–orbit interaction and the electron correlations is sensitive to different calculation methods (see Supplementary Note 4; Supplementary Figs 1 and 9).

Another possible cause of the discrepancy between the data and calculations is the strong coupling of electrons to the bosonic modes, which also could significantly renormalize the band shape. Indeed, the peak–dip–hump shape, which is a characteristic signature of strong mode coupling, is seen in EDCs (Fig. 3e), being consistent with this scenario. One of candidates for the bosonic modes is the phonons, which are usually coupled to the correlated systems very strongly. Another candidate is suggested by the similarity of the slightly distorted band shape to measurements in graphene, where it was attributed to electron–plasmon coupling^24^. Namely, the origin could be the same in the two cases: emission of collective modes through vertical interband transitions becomes possible above a threshold energy, modifying the spectrum.

### Correlation-driven anomalous temperature evolution

The strongly correlated feature of 5*d* electrons of Pr_2_Ir_2_O_7_ should be observed in the ARPES spectra. We find such a signature in the temperature evolution of the spectral shape. Figure 5a,b plots Fermi-function-divided EDCs at Γ (*hν*=10.5 eV) and off Γ (*k*_(111)_=0.31 Å^−1^ reached at *hν*=21.2 eV). The sharp peak clearly seen in the low-temperature spectra is strongly suppressed at elevated temperatures. The behaviour is clearly more marked than the thermal broadening effects observed in the other strongly collated systems such as the well-studied cuprates, which have the ‘marginal' Fermi liquid state^25^,^26^. We note that the peak suppression in the present data is accelerated across ∼100 K, differently from the typical thermal broadening with a gradual increase over a wider temperature range. We find that, associated with the suppression, a large portion of spectral weight above *E*≃−0.1 eV is transferred to higher binding energies in Pr_2_Ir_2_O_7_. A plausible origin for it is the polaronic effects, which could become crucial in the purely screened electronic systems, as also proposed for the other strongly correlated systems such as the perovskite iridates, manganites and lightly doped cuprates^27^,^28^,^29^. These features are compatible with the non-Fermi liquid behaviour predicted theoretically for the Fermi node phase in refs 11, 30, and may also be related to recent observations of quantum critical behaviour in thermodynamic measurements of Pr_2_Ir_2_O_7_ (ref. 17). However, a fuller identification of the non-Fermi liquid state and explication of its physics in Pr_2_Ir_2_O_7_ requires higher resolution data and more elaborate analysis, beyond the scope of this paper.

### Quadratic band touching

In passing, we point out that broad spectral weight emerges beyond *E*_F_ as seen in the Fermi-function-divided image for the 75-K data (arrow in Fig. 5f). The related spectral intensity is obtained off Γ, showing an upturn behaviour beyond *E*_F_ (arrow in Fig. 5b and Supplementary Figs 10 and 11, and see Supplementary Notes 5 and 6). While the strong suppression of quasiparticle peaks at elevated temperatures prevents us from a definitive determination of the conduction band dispersion, the observation of spectral weight above *E*_F_ is compatible with the predicted existence of a quadratic band touching on the unoccupied side (Fig. 5d).

## Discussion

Prior measurements^6^,^16^,^31^ showing ferromagnetic spin-ice-type correlations among the Pr moments below 2 K may be explained due to unconventional ferromagnetic RKKY interactions arising from the point-like Fermi surface. The Fermi node also leads to strong sensitivity to small time-reversal breaking perturbations, producing Weyl points close to the Fermi energy^12^ and a gigantic anomalous Hall effect. This is also in accord with the experimental fact that Pr_2_Ir_2_O_7_ was the first material found to exhibit a large spontaneous Hall effect in a spin liquid state at zero field^6^,^31^.

Our results suggest that tuning of a unique quantum critical point between an antiferromagnetic Weyl semimetal and the non-Fermi liquid nodal phase may be possible by alloying or hydrostatic pressure. Correlated topological phases and device applications with iridate films could be accessed by controlling the strain-induced breaking of the cubic crystal symmetry and size quantization (sub-band formation) in quantum well structures. We indeed verified theoretically the opening of a significant topological gap with uniaxial compression along the 〈111〉 axis by first-principles calculations (see Supplementary Fig. 12 and Supplementary Note 7). This analysis, moreover, shows the presence of three two-dimensional surface Dirac cones in this TI state (see Supplementary Fig. 13; Supplementary Table 1; Supplementary Note 7). It will be exciting to investigate whether correlations, neglected in the density function theory, lead to spontaneous time-reversal breaking at surfaces^32^,^33^ with fractional excitations^34^, or surface topological order^35^,^36^,^37^,^38^, both of which have been predicted theoretically.

## Methods

### Samples and ARPES experiments

Single crystals of Pr_2_Ir_2_O_7_ with 1 mm^3^ size were grown using a flux method. The sample surface with the (111) plane was prepared by cleaving the single crystal *in situ* with a top post clued on the crystal.

The ARPES experiments were performed at BL7U of Ultraviolet Synchrotron Orbital Radiation (UVSOR) facility (*hv*=8∼18 eV) with a MBS A-1 electron analyzer^23^, BL28A of Photon Factory, KEK (*hv*=39∼60 eV) with a Scienta SES2002 electron analyzer and in our laboratory using a system consisting of a Scienta R4000 electron analyzer equipped with a 6.994-eV laser (the 6th harmonic of Nd:YVO_4_ quasi-continuous wave with a repetition rate of 240 MHz)^22^ and He discharge lamp (HeI*α*, *hν*=21.2 eV). The overall energy resolution in the ARPES experiment was set to ∼2 meV and ∼6 meV in the lab system with a laser and He lamp, respectively, and ∼15 meV for the synchrotron facility data. The sample orientation was determined with a Laue picture taken before the ARPES experiment.

The intrinsic *k*_*z*_ broadening, *δk*_*z*_, is inversely proportional to the photoelectron escape depth *λ* (*δk*_*z*_=1/*λ*). We used low photon energies to find the nodal point in the 1st BZ, which enables the bulk sensitive measurements. For example, the photon energy corresponding to the Γ point is around 10 eV, at which the *λ*-value is estimated to be ∼20 Å according to the ‘universal curve'. It translates into *δk*_*z*_ with ∼5 % of the BZ size, which is rather small and sufficient to validate our results. In the Supplementary Fig. 4, we demonstrate that the *δk*_*z*_ (or *δk*_(111)_) is small enough to resolve the *E* versus *k*_(111)_ relation with a 1-eV step of photon energy. We also used higher photon energies of ∼50 eV to investigate the 3rd BZ. In this case, the *λ*-value becomes small (∼5 Å), resulting in a relatively large *δk*_*z*_ (∼20 % of the BZ). Nevertheless, we note that the energy dispersion in Pr_2_Ir_2_O_7_ is very weak especially around the Γ point (quadratic Fermi node), thus the effects of *k*_*z*_ broadening on the quasiparticle peaks should not be critical in our study.

### Band calculations

Calculations were carried out using both the standard generalized gradient approximation (GGA) and the Tran–Blaha modified Becke–Johnson (TB-mBJ)^39^ exchange potential as implemented in Wien2k, for a series of different A-site ions. An RK_max_ parameter 7.0 was chosen and the wavefunctions were expanded in spherical harmonics up to *l*_max_^wf^=10 inside the atomic spheres and *l*_max_^pot^=4 for non-muffin tins. Bulk A_2_Ir_2_O_7_ (A=rare-earth element) has a cubic crystal structure with the space group Fdm. The experimental lattice parameters were used for A=Pr, Nd, Eu and Y in both GGA and TB-mBJ paramagnetic calculations, and spin–orbit coupling was applied to both the heavy rare-earth element and the Ir electrons. The paramagnetic GGA and TB-mBJ calculations put the 4f states of the rare-earth element at the Fermi energy; to avoid this, since the 4f electrons are highly localized, their potential is shifted by a constant.

The TB-mBJ method is believed to produce improved results for small band gap systems^40^, and has been widely used in studies of topological insulators^41^. Both methods (GGA and TB-mBJ) yield almost indistinguishable results away from the Fermi energy, with a slight decrease of bandwidth in the TB-mBJ calculation, and small differences near *E*_*F*_, as shown in Supplementary Fig. 1. We observed the symmetry-required nodal band touching at Γ for all calculations, but the Fermi energy is shifted from the nodal point by an amount that decreases with increasing A (A=Y, Eu, Nd and Pr) site ionic radius in both methods^42^. This occurs because of accidental crossing of states near the L point, where the valence band rises and approaches the Fermi energy, especially in the smaller rare earths. In general, the GGA calculation underestimates the effects of correlations, which tend to narrow the bands and to push occupied states deeper below the Fermi energy. Hence, our expectation is that the accidental crossing near L, which is responsible for the Fermi level shift in GGA, will be suppressed by correlations. The TB-mBJ method may be regarded as a crude way to do this. Indeed, in the presumed more accurate TB-mBJ method, all the paramagnetic band structures for the A_2_Ir_2_O_7_ series show smaller shifts of the Fermi level compared with their GGA counterparts. In the case of A=Pr, we find the shift vanishes and the nodal point occurs precisely at the Fermi energy. While there is a universal trend of a smaller shift of the Fermi energy as rare-earth ionic radius increases for the rare-earth elements we have tested, the small differences between GGA and TB-mBJ suggest that some theoretical uncertainties remain. We note, however, that we expect methods that include correlations more accurately, such as combined local density approximation and dynamical mean field theory (LDA+DMFT), are likely to further suppress the states near the Fermi energy at L even beyond TB-mBJ, favouring placing the Fermi level precisely at the node.

## Additional information

**How to cite this article:** Kondo, T. *et al.* Quadratic Fermi node in a 3D strongly correlated semimetal. *Nat. Commun.* 6:10042 doi: 10.1038/ncomms10042 (2015).

## Supplementary Material

Supplementary Information Supplementary Figures 1-13, Supplementary Table 1, Supplementary Notes 1-7 and Supplementary References.

## Figures and Tables

**Figure 1:**
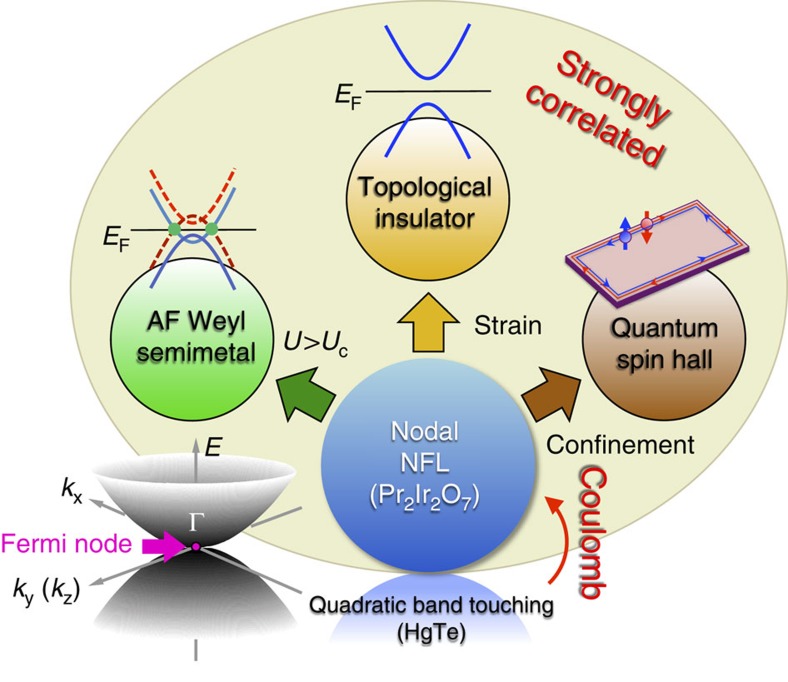
Quadratic Fermi node state of Pr_2_Ir_2_O_7_ tunable into interacting topological phases. In the lower part of the diagram, the bottom half of the blue circle and its reflection form a caricature of the quadratically dispersion conduction and valence bands touching at the zone centre, while at the same time the darker-blue upper circle suggests how Pr_2_Ir_2_O_7_, with non-negligible Coulomb interactions, is a strongly correlated non-Fermi liquid analogue of HgTe, shown as a pale-blue reflection. Arrows indicate the perturbations that convert the nodal non-Fermi liquid state to diverse topological phases: an antiferromagnetic Weyl semimetal should be produced in bulk materials by alloying or hydrostatic pressure, uniaxial strain yields a three-dimensional topological insulator and two-dimensional confinement produces a quantum spin Hall state. The outer circle reminds us that all the states produced in these ways retain strong correlations, and hence are excellent candidates for observing non-trivial surface phases different from those of band theory, as discussed in the text.
