# Supplementary material for: Quadratic Fermi node in a 3D strongly correlated semimetal
Source: Nat Commun. 2015 Dec 7;6:10042. doi: 10.1038/ncomms10042 (PMC4686656; doi:10.1038/ncomms10042)
Supplement: Supplementary Information — Supplementary Figures 1-13, Supplementary Table 1, Supplementary Notes 1-7 and Supplementary References. [file ncomms10042-s1.pdf]

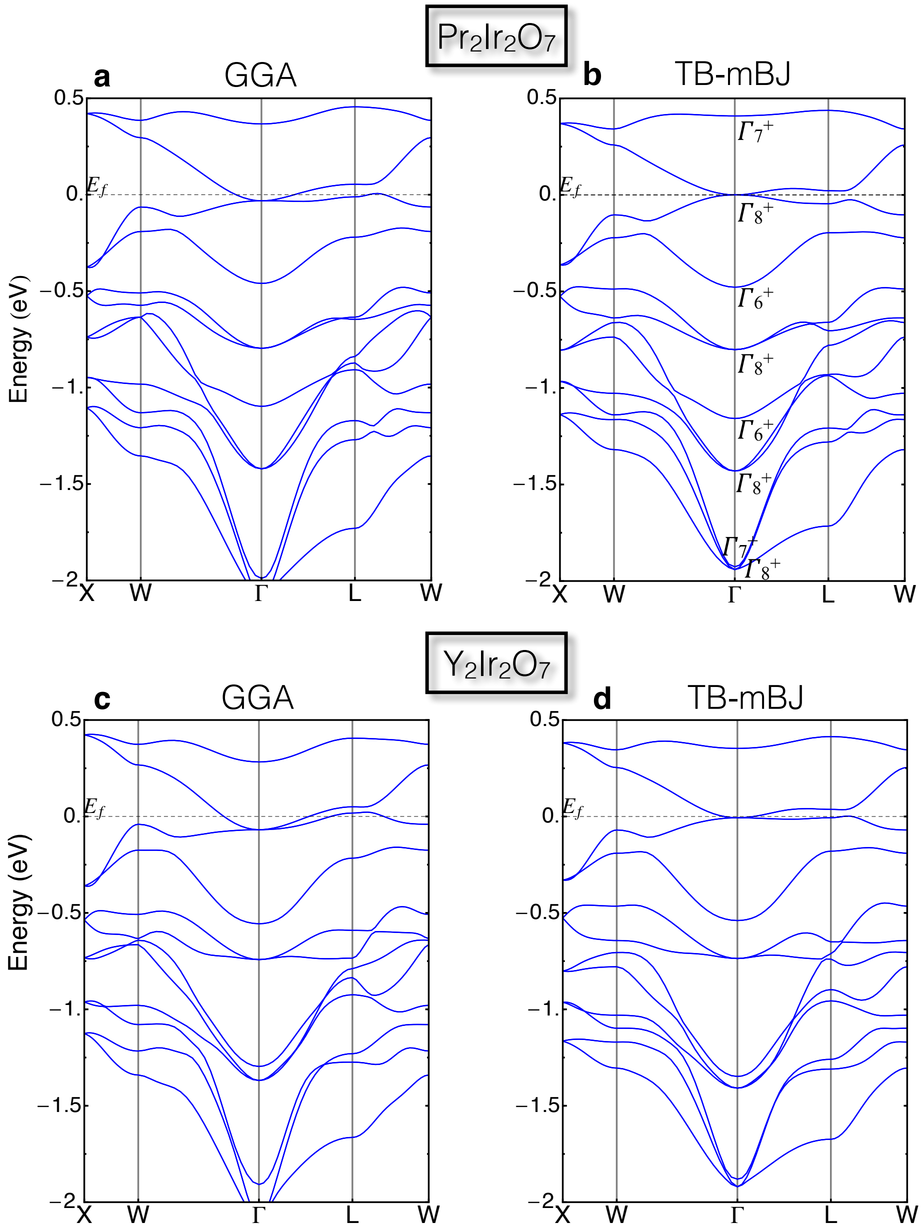

Supplementary Figure 1: **a,b,** Paramagnetic band structure within GGA and TB-mBJ, respectively, in the case of A=Pr. In **b**, the bands are identical to Fig. 2e, with irreducible representation group at the Γ point shown. The main band characters around the Fermi energy are of Ir *d* bands. **c,d,** Paramagnetic band structure within GGA and TB-mBJ, respectively, in the case of A=Y.

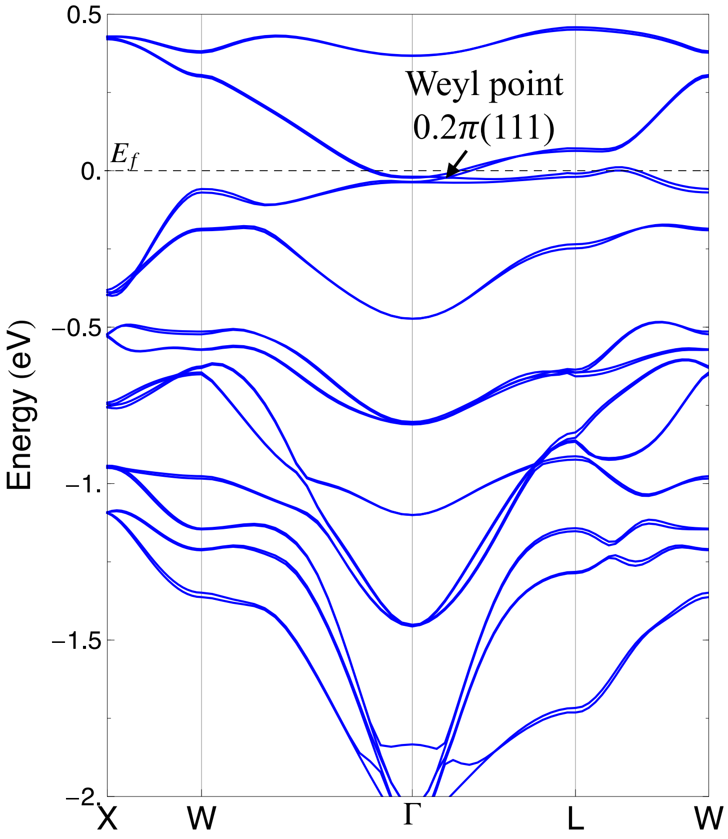

Supplementary Figure 2: The magnetic band structure calculation on $Pr_2Ir_2O_7$, with Pr $4f$ moment pointing along the all-in all-out direction.

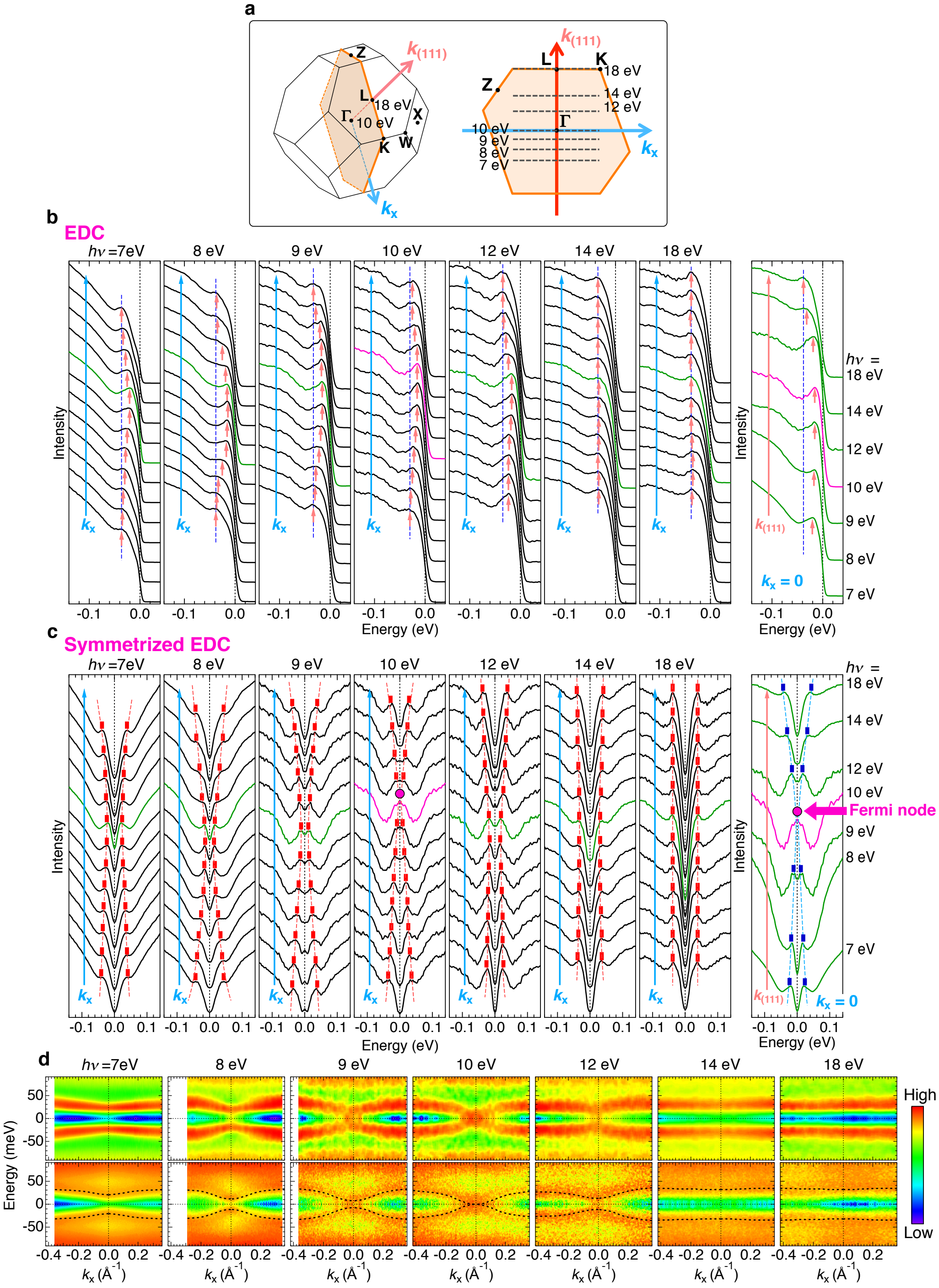

Supplementary Figure 3: **a**, Brillouin zone, indicating the measured momentum plane across Γ, L and K (painted by orange) and momentum cuts for various photon energies (dashed lines). **b**, Energy distribution curves (EDCs) measured by ARPES along k_x (defined in **a**). The used photon energy is described on the top of each panel, and the corresponding $k_{(111)}$ location is indicated in **a**. In the right panel (green and magenta curves), the EDCs at $k_x = 0$ are extracted. The magenta arrows mark the energy position of spectral peak. A blue dashed line is plotted in each panel to confirm an energy dispersion of the peaks. **c**, The symmetrized EDCs of **b**. The red bars and dashed curves indicate the spectral peaks and the energy dispersion, respectively. In the right panel (green and magenta curves), the curves at $k_x = 0$ are extracted. **d**, ARPES images symmetrized about E_F obtained at various photon energies (bottom panels), and the second derivative of each image (top panels). The measured momentum cuts are indicated in **a** with dashed lines.

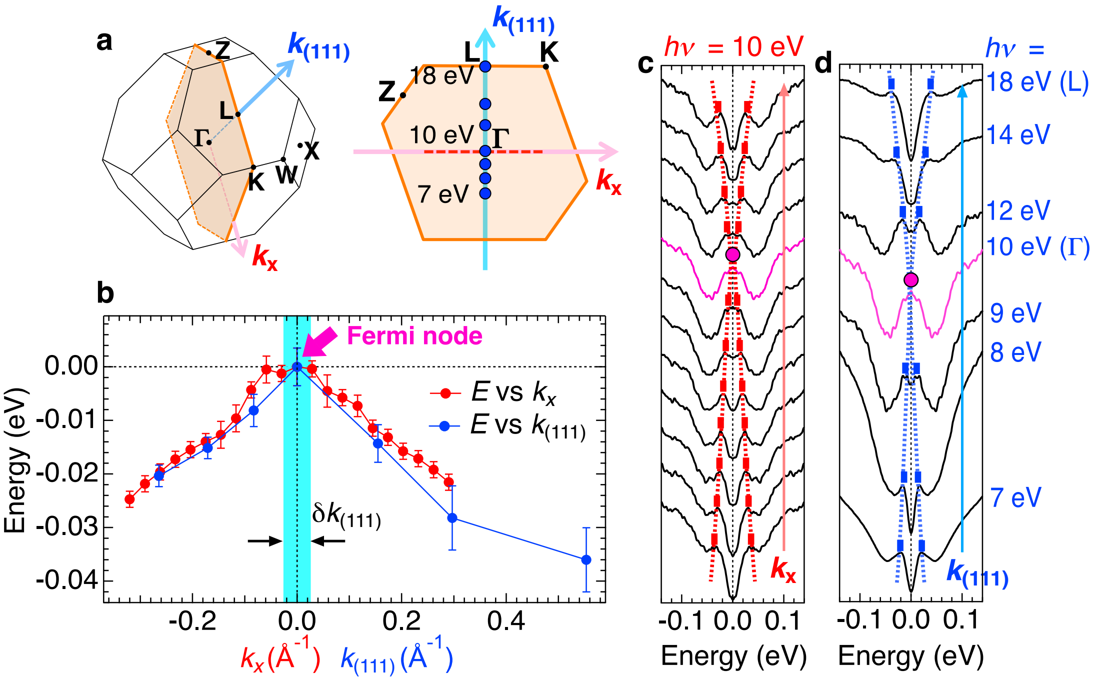

Supplementary Figure 4: Comparison between the energy dispersion along two directions [$k_{(111)}$ and perpendicular to it (k_x)], and an estimation of the k_z-broadening (δk_z or $\delta k_{(111)}$). **a**, Brillouin zone of $Pr_2Ir_2O_7$. **b**, The energy dispersions along the k_x and $k_{(111)}$ directions (red and blue circles, respectively), determined from the peak positions of spectra in **c** and **d**. The former is measured at $h\nu$=10eV (red dashed line in the right panel of **a**), and the latter at various photon energies (blue circles in the right panel of **a**). The experimental k_z broadening (δk_z or $\delta k_{(111)}$) is estimated to be $\sim$ 5% of Brillouin zone at $h\nu$=10eV (Γ point), which is indicated with a light blue belt and black dimension arrows. **c**, **d** The ARPES spectra (symmetrized EDCs) along k_x and $k_{(111)}$, respectively. Error bars in **b** represent uncertainty in estimating the spectral peak positions.

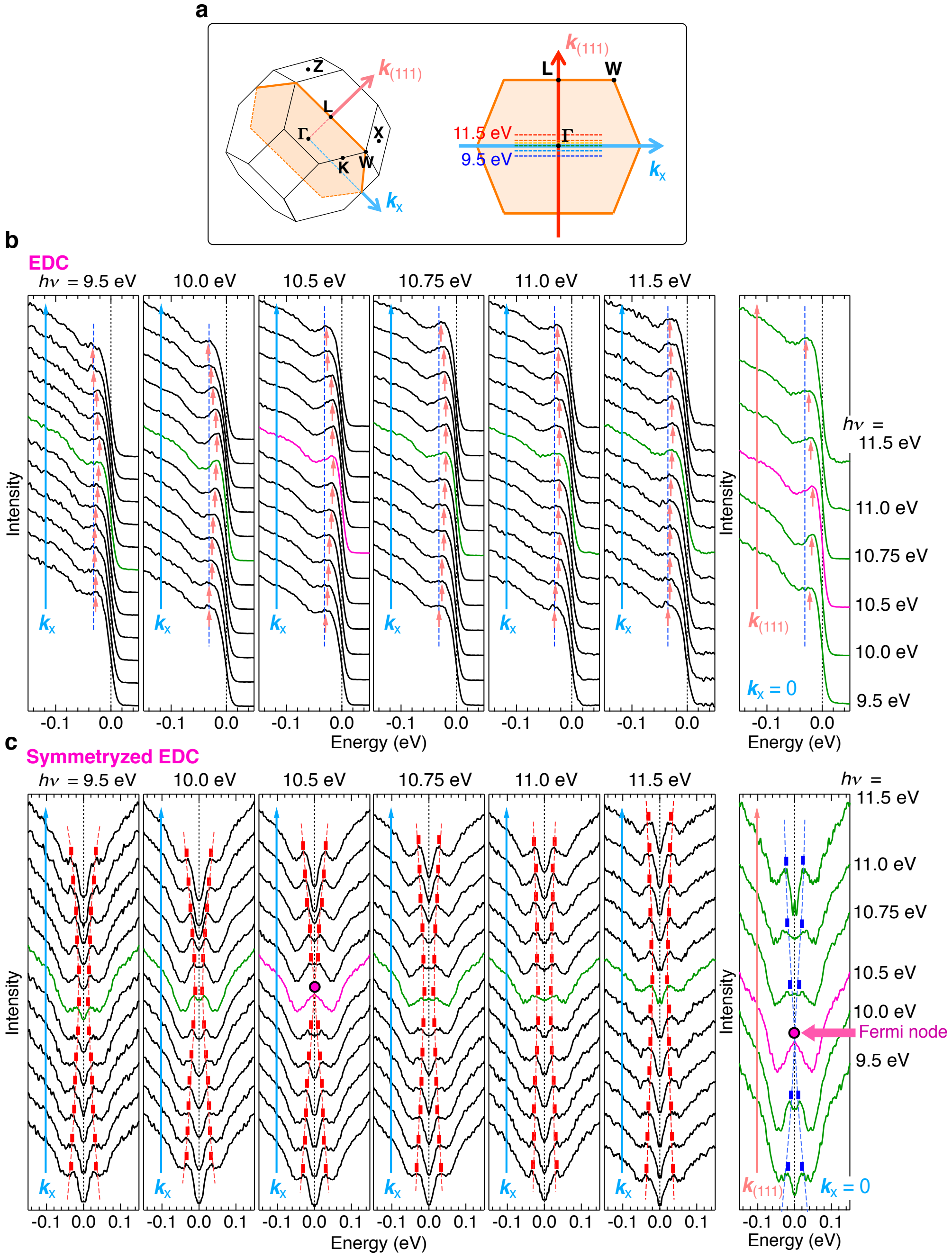

Supplementary Figure 5: **a**, Brillouin zone, indicating the measured momentum plane across Γ, L and W (painted by orange) and momentum cuts for various photon energies (dashed lines). **b**, Energy distribution curves (EDCs) measured by ARPES along k_x (defined in **a**). The used photon energy is described on the top of each panel, and the corresponding $k_{(111)}$ location is indicated in **a**. In the right panel (green and magenta curves), the EDCs at $k_x = 0$ are extracted. The magenta arrows mark the energy position of spectral peak. A blue dashed line is plotted in each panel to confirm an energy dispersion of the peaks. **c**, The symmetrized EDCs of **b**. The red bars and dashed curves indicate the spectral peaks and the energy dispersion, respectively. In the right panel (green and magenta curves), the curves at $k_x = 0$ are extracted.

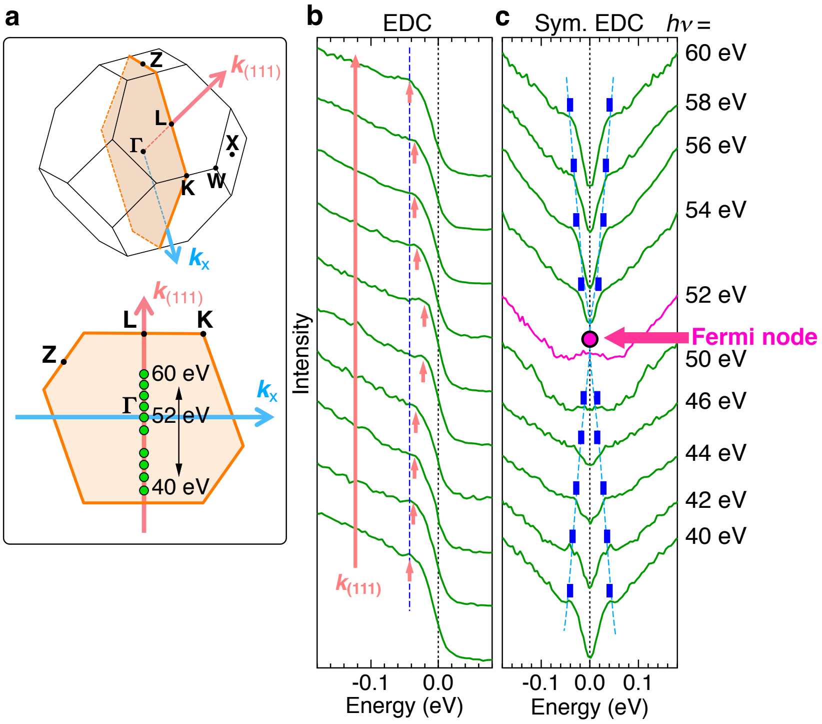

Supplementary Figure 6: **a**, Brillouin zone of $Pr_2Ir_2O_7$, indicating the measured momentum plane (painted by orange) and momentum points for various photon energies (green circles). **b**, EDCs at various momentum points along $k_{(111)}$ (green circles in **a**) measured at different photon energies. The magenta arrows indicate the peak positions of the spectra. A blue dashed line is added to confirm an energy dispersion of the peaks along $k_{(111)}$. **c**, The symmetrized EDCs of **b**. The blue bars and dashed curves indicate the spectral peaks and the energy dispersion, respectively.

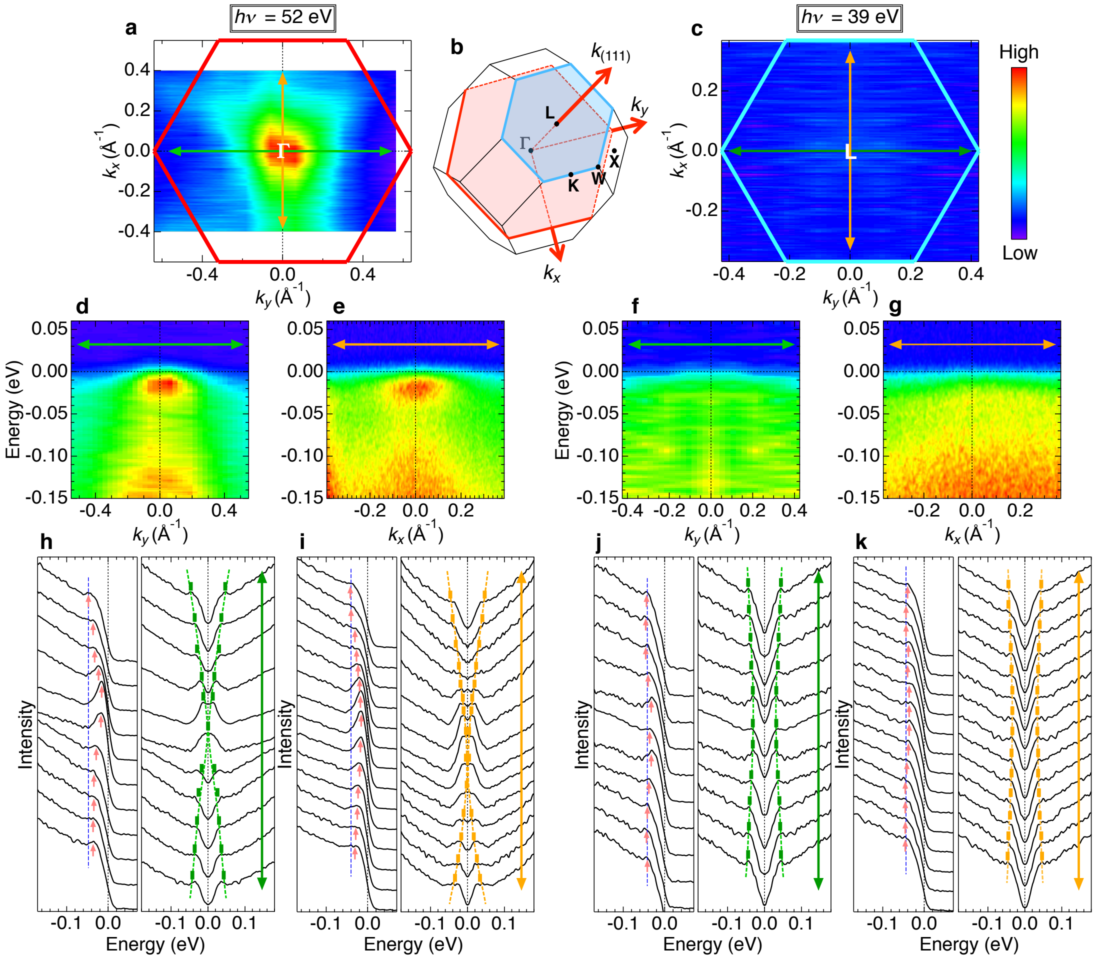

Supplementary Figure 7: **a**, **c**, ARPES intensities at E_F along momentum sheets perpendicular to the $k_{(111)}$ axis (see panel **b**), crossing Γ ($h\nu = 52$ eV) and L ($h\nu = 39$ eV), respectively. For the data at $h\nu = 39$ eV, only the positive side of k_y were measured, and it is reflected to the negative side. **b**, Brillouin zone, indicating the momentum sheets for the data of **a** and **c**, which are painted by red and blue, respectively. **d**, **e**, Band dispersion maps along k_y and k_x marked by a green and orange arrows in **a**, respectively. **f**, **g**, Band dispersion maps along k_y and k_x marked by a green and orange arrows in **c**, respectively. **h**, **i**, The left panels plot EDCs extracted from **d** and **e**, respectively. The magenta arrows indicate energies of spectral peaks. A blue dashed line is added to confirm the energy dispersion. To the right, the symmetrized EDCs are shown. Green and orange bars and dashed curves indicate the spectral peaks and the energy dispersion of those. **j**, **k**, The same data as **h** and **i**, but extracted from **f** and **g**, respectively.

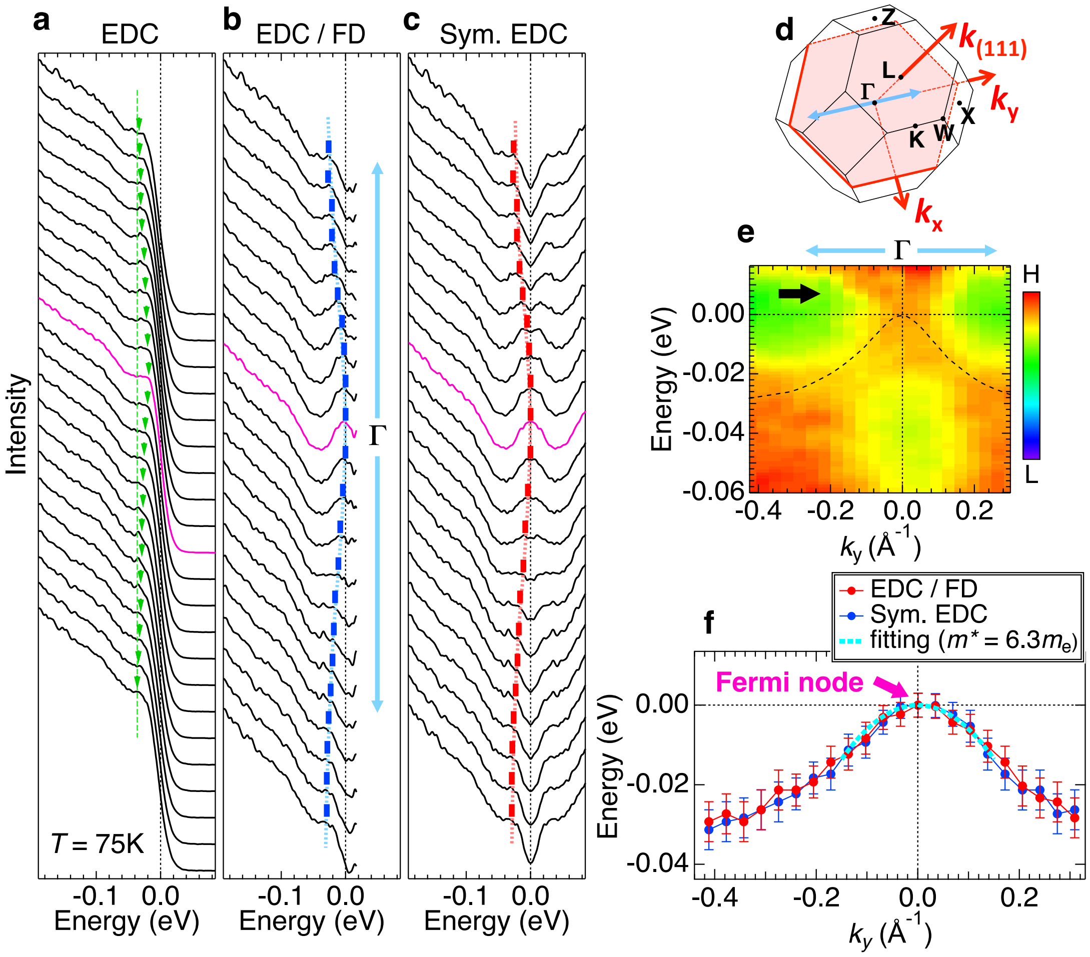

Supplementary Figure 8: Consistency between the results obtained with the FD-division method and the symmetrization method. **a**, The ARPES spectra (EDCs) measured at $T = 75$K along a momentum cut crossing Γ indicated in **d** with a light blue arrow. The green dashed line is a guide to eyes for a confirmation of dispersion in the peaks (green arrows). **b**, The same EDCs in **a**, but divided by the Fermi function at 75K convoluted with the experimental energy resolution. **c**, The same EDCs in **a**, but symmetrized about E_F. The color bars and dashed curves in **b** and **c** indicate the spectral peaks and the energy dispersions, respectively. **d**, Brillouin zone of $Pr_2Ir_2O_7$ with a measured momentum cut (a light blue arrow). **e**, The corresponding ARPES image after the treatment of FD-division. The black arrow points to the remarkable intensity above E_F, implying the existence of conduction band touching on the unoccupied side. **f**, The band dispersion extracted from the peak positions of spectra in **b** and **c**. The light blue dashed curve is a quadratic dispersion fitted to the data. Error bars in **f** represent uncertainty in estimating the spectral peak positions.

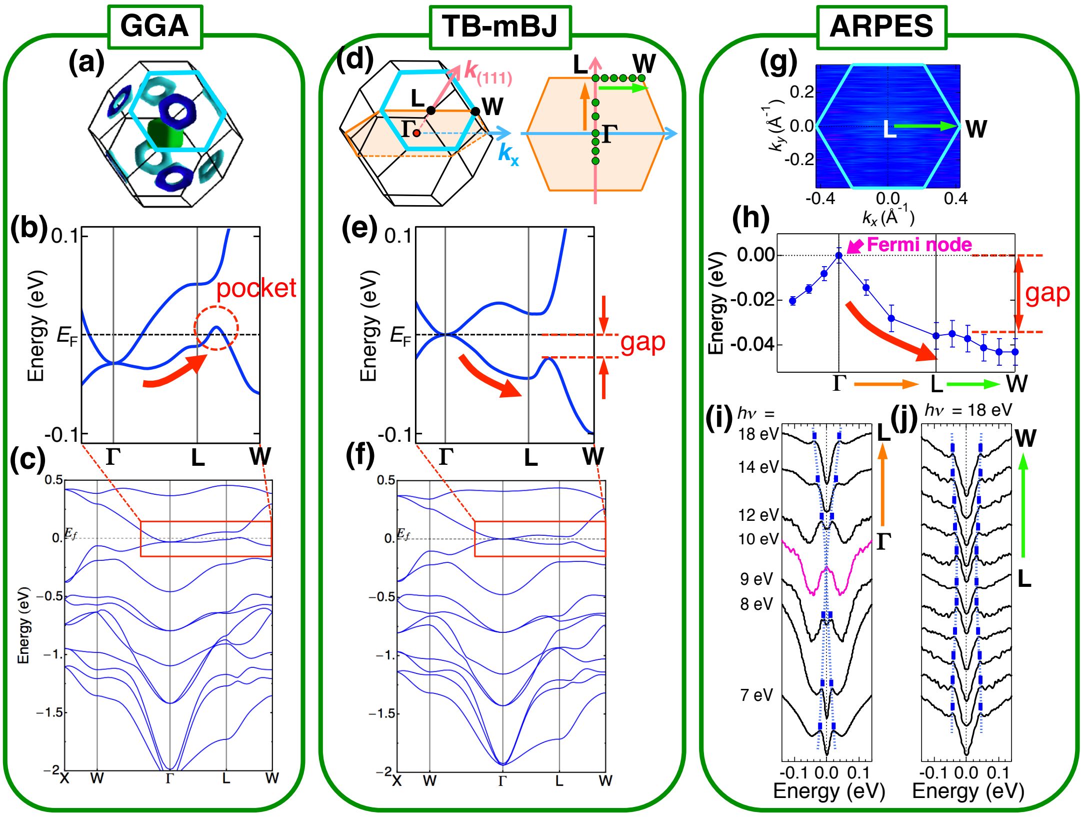

Supplementary Figure 9: Agreement of the ARPES data with the TB-mBJ calculation, and disagreement with the GGA calculation. **a-c,** The results of GGA calculations: the Fermi surfaces (**a**) and the energy dispersions (**b**, **c**). The magnified image of red square region in **c** is exhibited in **b**. **d-f,** The results of TB-mBJ calculations: the Fermi node state (**d**) and the energy dispersions (**e**, **f**). The magnified image of red square region in **f** is exhibited in **e**. **g-j,** The ARPES results: the Fermi intensity along the $k_x - k_y$ plane crossing the L point (**g**) (light blue region marked in **a** and **d**), the energy dispersions determined along the high symmetry lines of Γ-L-W (**h**), and the corresponding ARPES spectra (symmetrized EDCs) along Γ-L (**i**) and L-W (**j**). The observed k points are marked in the right panel of **d** with green circles. The band dispersion obtained by GGA calculation approaches E_F from Γ toward L (tick red arrow in **b**), and crosses E_F along the L-W cut, generating a Fermi pocket (red dashed circle in **b**). These features disagree with the ARPES data, demonstrating that the band disperses toward higher binding energies along the Γ-L cut with a gap opening all the way toward the W point without crossing E_F. On the other hand, the TB-mBJ calculation reproduces all the features seen in the ARPES data pointing to the quadratic Fermi node state. Error bars in **h** represent uncertainty in estimating the spectral peak positions.

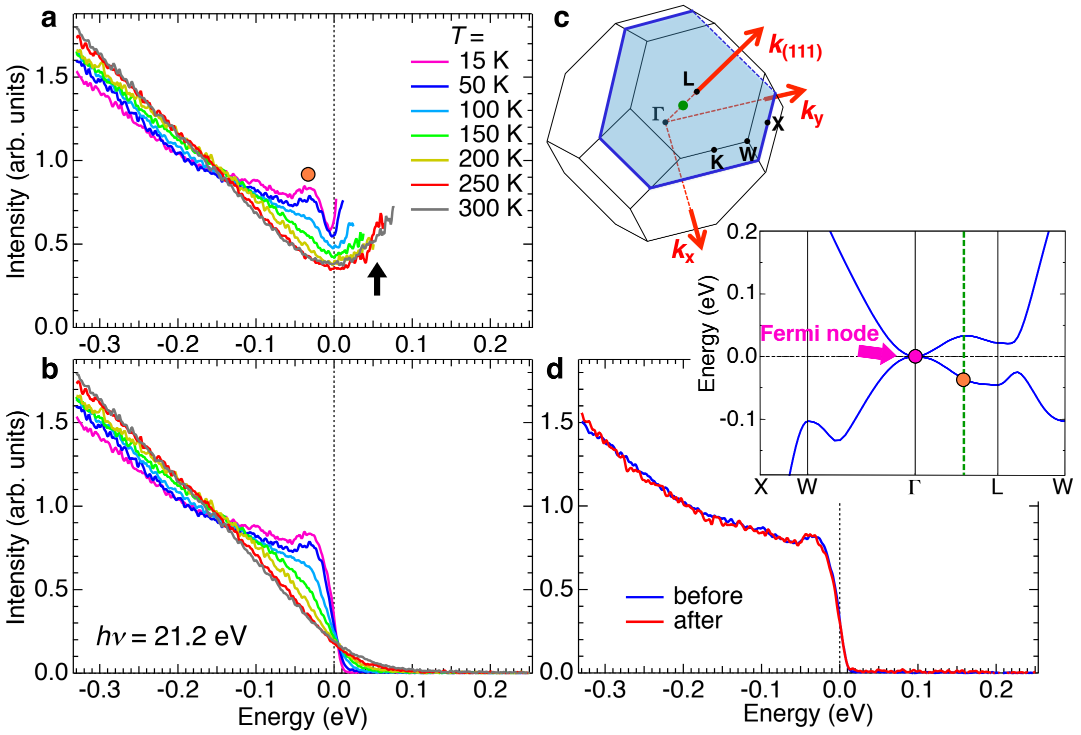

Supplementary Figure 10: Dramatic peak suppression at elevated temperatures, and aging check after the measurement of temperature sweeping. **a**, Temperature evolution of EDCs divided by the energy-resolution convoluted Fermi function at measured temperatures. The 21.2 eV photons (HeIα) were used, and the observed k point is marked in **c** with a green circle, and also in the inset of **d** with a green dashed line. The peak position is marked by an orange circle. The black arrow in **a** indicates an upturn behavior of the spectra beyond E_{F}. The data up to 150K are shown in Fig. 5 of the main paper. Here we show the data up to the higher temperature of 300K. **b**, EDCs used for the analysis in **a**. **c**, Brillouin zone: a momentum sheet measured at $h\nu = 21.2$ eV and the k point where EDCs of **a** were taken are marked by a blue sheet and a green circle, respectively. **d**, The following two energy distribution curves are compared; one taken just after the sample cleaving and the other taken after completing the temperature scan presented in **b**. These two curves almost perfectly overlap, signifying that there is no aging of sample surface during the entire measurement. The inset shows the calculated band dispersion along the high symmetry lines (the same figure as Fig. 5d).

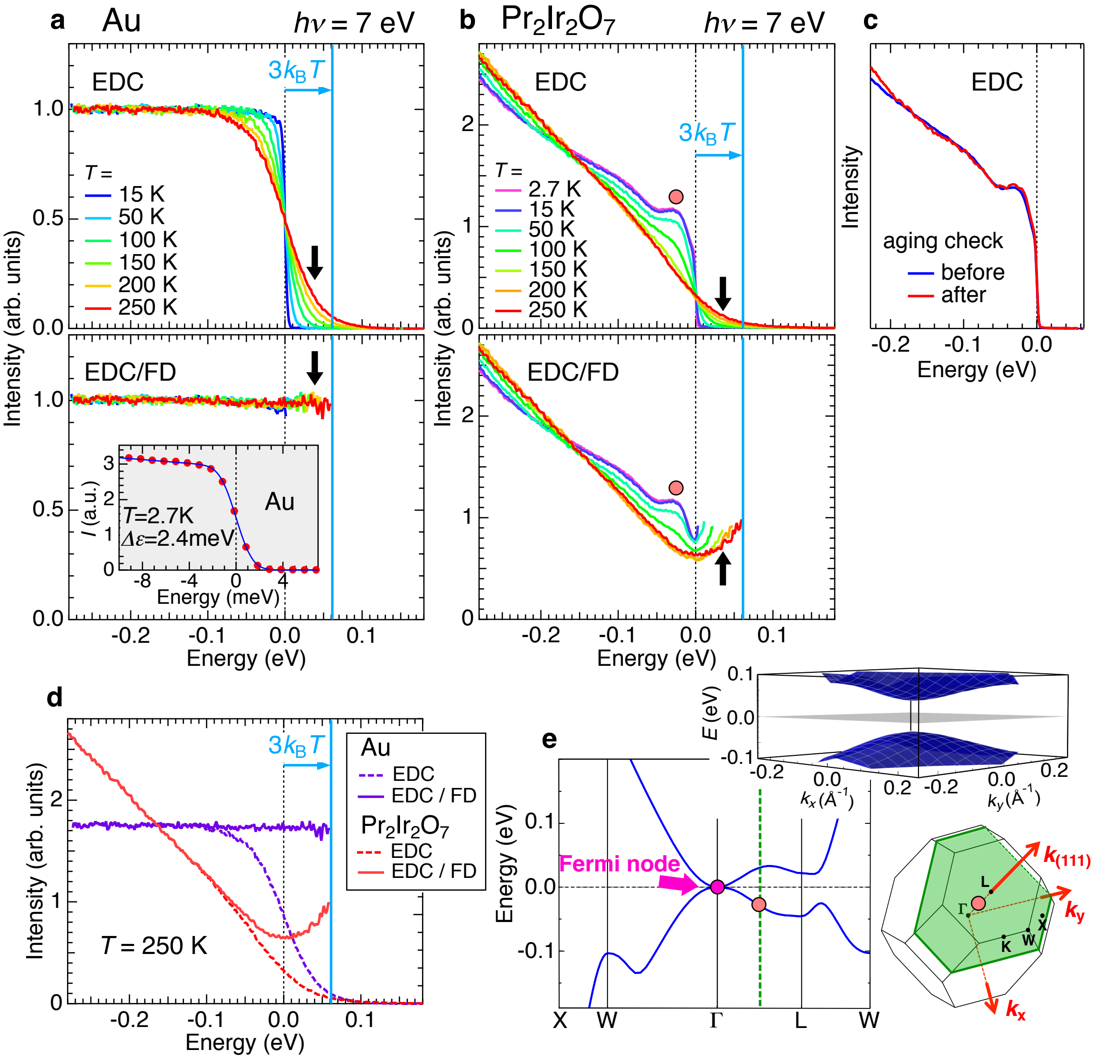

Supplementary Figure 11: Signature of the unoccupied conduction band of $Pr_2Ir_2O_7$ observed by a laser ARPES with ultra-high energy resolution. **a**, The ARPES spectra of Au (electrically contacted to the samples) measured at various temperatures by a 7-eV laser ARPES (the upper panel). In the bottom panel, the same spectra, but divided by the Fermi function at the measured temperature convoluted with the experimental energy resolution, are plotted. The energy resolution is estimated to be $\Delta\varepsilon = 2.4$ meV in the present experimental setting as demonstrated in the inset panel using the Au spectrum at the lowest temperature ($T = 2.7$K). **b**, Temperature evolution of ARPES spectra for the sample (the upper panel) and the same curves after the FD-division (the lower panel), measured at the midpoint between the Γ and L points reached at $h\nu = 7$eV. The experimental setting and the FD-division method applied to the data are exactly the same as for the Au in **a**. The arrows in **a** and **b** mark the thermally populated spectral weight above E_F, exhibited up to $3k_BT$. **c**, The comparison of spectral shape before and after the temperature scan. **d**, Comparison between the spectra of Au and the sample (EDCs and FD-divided EDCs extracted from **a** and **b**). **e**, Band calculation close to E_F. The k_z (or $k_{(111)}$) value at $h\nu = 7$eV is located at the midpoint between the Γ and L points (a dashed green line and a green $k_x - k_y$ plane).

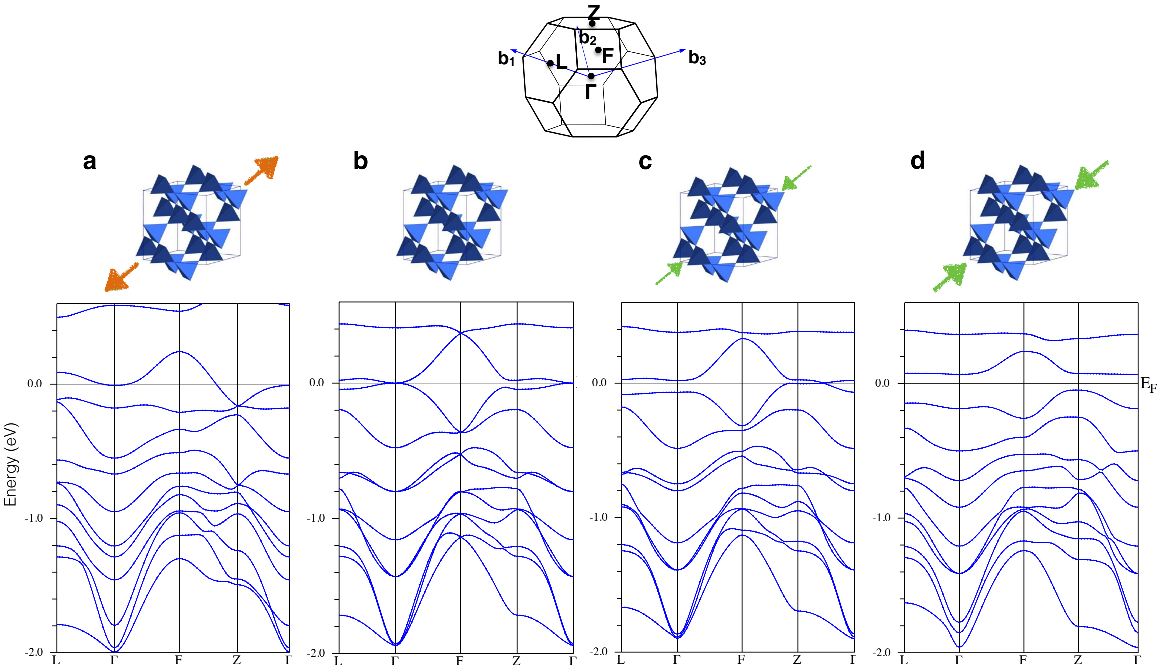

Supplementary Figure 12: Schematic graphs of $Pr_2Ir_2O_7$ under uniaxial pressure along $\langle 111 \rangle$ direction and the corresponding Brillouin zone of the rhombohedral unit cell, with TRIM specified. **a,** TB-mBJ paramagnetic band structure of $Pr_2Ir_2O_7$ under 5 % in-plane compressive strain ($a = 0.95a_0$, $a_0 = 7.35$ Å, the bulk in-plane lattice parameter in the hexagonal cell). The band crossing occurs along $\langle 111 \rangle$ direction, which is located at the Z point (π,π,π). **b,** TB-mBJ paramagnetic band structure of $Pr_2Ir_2O_7$ bulk in the absence of pressure or strain in the rhombohedral unit cell. **c,** TB-mBJ paramagnetic band structure of $Pr_2Ir_2O_7$ under 1 % in-plane tensile strain ($a = 1.01a_0$). A tiny gap, 4 meV is opened, which can not be oberved from the scale shown. **d,** TB-mBJ paramagnetic band structure of $Pr_2Ir_2O_7$ under 5 % in-plane tensile strain ($a = 1.05a_0$). The gap is clearly shown.

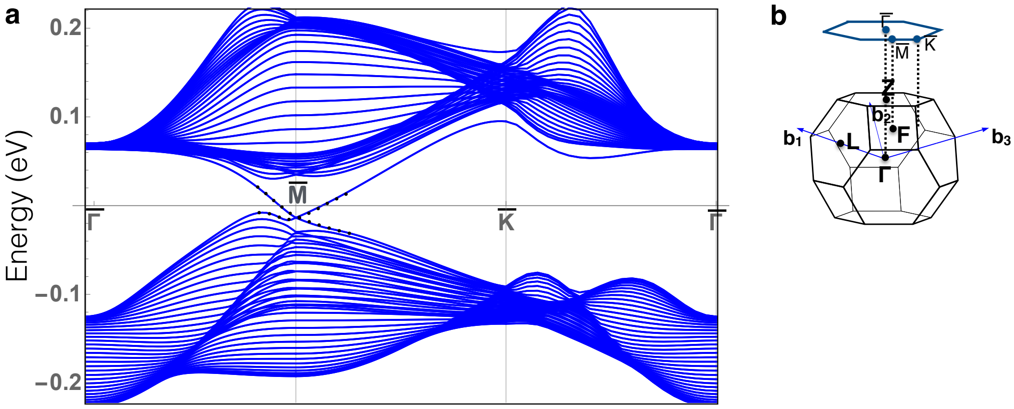

Supplementary Figure 13: **a,** The paramagnetic band structure of $Pr_2Ir_2O_7$ under 5% uniaxial strain on the $\langle 111 \rangle$ surface. The topological surface state is marked with dotted lines and the Dirac cones are located at the $\overline{\mathrm{M}}$ point. **b,** The surface 2D Brillouin zone is shown by the blue hexagon, in which the high symmetric k points $\overline{\Gamma}$, $\overline{\mathrm{M}}$ and $\overline{\mathrm{K}}$ are labeled.

Γ	L	Z	F
+1	-1	-1	-1

Supplementary Table 1: Parity eigenvalues at the TRIM for $Pr_2Ir_2O_7$ under uniaxial pressure along $\langle 111 \rangle$.

Supplementary Note 1: Paramagnetic Band Calculations for cubic $A_2Ir_2O_7$

In this supplemental section, we discuss the electronic structure calculations in the paramagnetic state. Calculations were carried out using both the standard generalized gradient approximation (GGA) [1] and the Tran-Blaha modified Becke-Johnson (TB-mBJ) [2] exchange potential as implemented in Wien2k [3], for a series of different A site ions. An RKmax parameter 7.0 was chosen and the wave functions were expanded in spherical harmonics up to $l^{wf}_{max} = 10$ inside the atomic spheres and $l^{pot}_{max} = 4$ for non-muffin tins. Bulk $A_2Ir_2O_7$ (A=rare earth element) has a cubic crystal structure with the space group Fd$\bar{3}$m. The experimental lattice parameters [4–7] were used for A= Pr, Nd, Eu and Y in both GGA and TB-mBJ paramagnetic calculations, and spin-orbit coupling was applied to both the heavy rare earth element and the Ir electrons. The paramagnetic GGA and TB-mBJ calculations put the 4f states of the rare earth element at the Fermi energy; to avoid this, since the 4f electrons are highly localized, their potential is shifted by a constant.

The TB-mBJ method is believed to produce improved results for small band gap systems [8], and has been widely used in studies of topological insulators [9]. Both methods yield almost indistinguishable results away from the Fermi energy, with a slight decrease of band-width in the TB-mBJ calculation, and small differences near E_F, as shown in Supplementary Fig. **1**. We observed the symmetry-required nodal band touching at Γ for all calculations, but the Fermi energy is shifted from the nodal point by an amount that decreases with increasing A (A= Y, Eu, Nd and Pr) site ionic radius in both methods [10]. This occurs because of accidental crossing of states near the L point, where the valence band rises and approaches the Fermi energy, especially in the smaller rare earths. For example, a small shift of the Fermi level of about 30 meV in the GGA approximation is observed in $Pr_2Ir_2O_7$, while the shift is about 70 meV in $Y_2Ir_2O_7$. In general, the GGA calculation underestimates the effects of correlations, which tend to narrow the bands and to push occupied states deeper below the Fermi energy. Hence our expectation is that the accidental crossing near L, which is responsible for the Fermi level shift in GGA, will be suppressed by correlations. The TB-mBJ method may be regarded as a crude way to do this. Indeed, in the presumed more accurate TB-mBJ method, all the paramagnetic band structures for the $A_2Ir_2O_7$ series show smaller shifts of the Fermi level compared to their GGA counterparts. In the case of A=Pr, we find the shift vanishes and the nodal point occurs precisely at the Fermi energy, and a shift value of around 7 meV is observed in the case of A=Y. While there is a universal trend of smaller shift of the Fermi energy as rare earth ionic radius increases for the rare earth elements we have tested, the small differences between GGA and TB-mBJ suggest that some theoretical uncertainty remains. We note, however, that we expect methods that include correlations more accurately, such as LDA+DMFT, are likely to further suppress the states near the Fermi energy at L even beyond TB-mBJ, favoring placing the Fermi level precisely at the node.

The above calculation treats Pr as non-magnetic by artificially shifting the levels away from the Fermi energy. More properly, these levels are highly localized and, due to large Coulomb repulsion, form local moments, which fluctuate thermally in the range of the ARPES measurements. A calculation which includes the localized but thermally fluctuating paramagnet state of the Pr spins requires Dynamical Mean Field Theory (DMFT), which is beyond the scope of this paper. However, we expect that the Pr moments are in any case so strongly localized that they couple only weakly to the Ir electrons. We can test this, and thereby gauge the magnitude of such effects, by studying the system at low temperature using the GGA+U technique. This treats the localization of the 4f moments but does not allow them to fluctuate. It has indeed been inferred that in $Pr_2Ir_2O_7$, the Pr moments becomes quasi-static and form some 2-in/2-out structure below 0.3K.

As the 2-in/2-out states are degenerate, we instead focus on the simpler all-in/all-out configuration of 4f moments. Although this is not the proper arrangement in $Pr_2Ir_2O_7$, it still shows the magnitude of the effects to be expected from Pr magnetic order, and specifically from time-reversal symmetry breaking. To include the non-collinear magnetism, the calculation is performed in WIENNCM (non-collinear magnetic version of the Wien2k package) [11]. We take U on the $4f$ electrons to be 6eV and U on the Ir $5d$ electrons to be 0eV. It is inevitable that the Ir $5d$ electrons acquire a small magnetic moment, through their interaction with the Pr ones. The largest Ir moment introduced in the GGA+U calculation is below $0.004\mu_B$. Supplementary Fig. **2** shows the magnetic band structure of $Pr_2Ir_2O_7$. Generally the structure of the Ir bands is very similar to that in the paramagnetic GGA calculation. This proves the weak coupling of Pr moments to the Ir electrons. However, there is indeed, as expected by symmetry, a small energy splitting of the bands in the GGA+U band structure, comparing to the originally degenerate bands in the GGA calculation. This is consistent with broken time reversal symmetry, which,

as expected from low energy $k \cdot p$ theory, leads to the appearance of Weyl points along the $\Gamma - L$ branch. The point appears near the Γ point, along the Γ–L line, as shown in Supplementary Fig. **2**. Note that the band bending between the L and W points is larger in the GGA+U calculation than in the TB-mBJ one, so a Fermi pocket appears in this calculation in this region in the GGA+U result.

Supplementary Note 2: Energy distribution curves (EDCs) used for the symmetrization analysis

In Fig. 3 of the main paper, we show the energy distribution curves (EDCs) symmetrized about $E_{\rm F}$ to remove the cut-off effect by Fermi function. Here we present the row EDCs used for the analysis (Supplementary Fig. **3**). Moreover, we show some data which are discussed but not presented in the main paper (Supplementary Fig. **5** and **6**).

Supplementary Fig. **3**b shows the EDCs measured at various photon energies from $h\nu = 7$ eV to 18 eV. The measured momentum cuts (dashed lines in Supplementary Fig. **3**a) are located in the $k_x - k_{(111)}$ plane crossing Γ, L, and K. As marked by magenta arrows, the quasiparticle peaks clearly disperse along k_x, and that at $k_y = 0$ (green curve in each panel) most approaches $E_{\rm F}$ at $h\nu = 10$ eV as examined in the right panel of Supplementary Fig. **3**b. The symmetrized EDCs of Supplementary Fig. **3**b are plotted in Supplementary Fig. **3**c. (The same data at $h\nu = 7$, 8, 9, and 10 eV are shown in Figs. 3d of the main paper.) It is clearly seen that the quadratic dispersion approaches $E_{\rm F}$ with increasing the photon energy, touches $E_{\rm F}$ at 10 eV, and move away from $E_{\rm F}$ again with a further increase of $h\nu$. This behavior is more clearly demonstrated in the right panel of Supplementary Fig. **3**c, where the curves at $k_x = 0$ are extracted. The corresponding ARPES images symmetrized at E_F are exhibited in the bottom panels of Supplementary Fig. **3**d. The upper panels plot the second derivatives of those. The systematic variation of dispersions with photon energy, including the Fermi node state at $h\nu = 10$ eV, are clearly seen.

We have further confirmed the existence of Fermi node by observing a different momentum sheet crossing Γ, L, and W points using another piece of sample. The $k_x - k_{(111)}$ plane and measured momentum cuts corresponding to used photon energies (9.5 eV to 11.5 eV) are indicated in Supplementary Fig. **5**a. Supplementary Fig. **3**b shows the EDCs along k_x obtained at different photon energies. The $h\nu$ value was swept by a fine step (≤ 0.5 eV, see dashed lines in Supplementary Fig. **5**a). The quasiparticle peak disperses along k_x, and most approaches $E_{\rm F}$ at ~ 10 eV as examined in the right panel of Supplementary Fig. **5**b. The corresponding symmetrized EDCs are plotted in Supplementary Fig. **5**c. We identify the band touching to $E_{\rm F}$ in the data at $h\nu = 10.5$ eV. This is more clearly presented in the right panel of Supplementary Fig. **5**c, where the curves at $k_x = 0$ are extracted. The broad shape of quasiparticle peak in $Pr_2Ir_2O_7$ does not allow us to determine the exact photon energy for the Γ point. Nevertheless, our data signify that the $Pr_2Ir_2O_7$ has a 3D band structure with a Fermi node theoretically predicted.

In order to further validate the 3D nature of band structure in $Pr_2Ir_2O_7$, we examined it with higher photon energies ($39 \leqslant h\nu \leqslant 60$ eV), which reach the 3rd Brillouin Zone in the $k_{(111)}$ direction. Supplementary Fig. **6**b plots the EDCs at various photon energies corresponding to momentum points marked by green circles in Supplementary Fig. **6**a. The curves are symmetrized in Supplementary Fig. **6**c to judge whether or not the dispersion touches $E_{\rm F}$. A gap clearly observed at 40 eV gets smaller with increasing $h\nu$, and it becomes negligible at 52 eV, where a broad single peak is obtained. With a further increase of $h\nu$, the gap opens again showing two peak structure in the spectra, being consistent with the Fermi nodal state. The energy dispersion along $k_{(111)}$ is consistent with that seen in the 1st Brillouin Zone observed with lower photon energies. The periodic trend over a wide range of photon energy ensures that our data provide the intrinsic 3D bulk band structure of $Pr_2Ir_2O_7$.

In the right- and left-side panels of Supplementary Fig. **7**, we show the band dispersion along two $k_x - k_y$ planes across Γ ($h\nu = 52$ eV) and L ($h\nu = 39$ eV), respectively. The ARPES intensities at $E_{\rm F}$ (top panels), the dispersion maps along k_x and k_y (middle panels), and the corresponding symmetrized EDCs (bottom panels) are also presented in Fig. 4 of the main paper. Here we plot the original EDCs before the symmetrization (left panels in Supplementary Figs. **7**h-**7**k). As marked by magenta arrows in each panel, the quasiparticle peaks are observed. Importantly, while the dispersion is clearly seen across the Γ point (Supplementary Fig. **7**h and **7**i), it is negligible across the L point (Supplementary Fig. **7**j and **7**k), which agrees to the Fermi node state.

Supplementary Note 3: Agreement between the results obtained with symmetrized

spectra and FD-divided spectra

In the main paper, we use two different techniques to remove the Fermi cut-off effect from the ARPES spectra (EDCs). One is the symmetrization method, which flips the EDC about E_F and add it to the original curve. This technique is widely used to visualize the opening of a gap, and also to determine the Fermi crossing point (k_F point). The method is easy to use and applicable even for the low temperature spectra with negligible intensity above E_F, thus it has been commonly used for a gap estimation in the superconducting and density-wave materials. A weakness of this method is that it requires the particle-hole symmetry, which is not for sure in the unknown materials like $Pr_2Ir_2O_7$. The more straightforward way of eliminating the Fermi cut-off effect would be to divide the EDCs by the Fermi function (FD) at measured temperature. For the realistic analysis, the function actually used is convoluted with the experimental energy resolution. However, the FD-division method, though conceptually straightforward, also has a difficulty; the high temperature data are required, since it utilizes the thermally populated intensity above E_F. Moreover, the high quality data with a high signal/noise ratio are essential in order to reliably reproduce the spectral shape close to E_F. Therefore, the best way to extract the band dispersion close to E_F is to apply both the analyses of the symmetrization and FD-division methods to the same data, and confirm the consistency between the two results.

In Supplementary Fig.**8**, we demonstrate that the results obtained with these two methods agree with each other, pointing to the realization of the quadratic Fermi nodal state in $Pr_2Ir_2O_7$. Supplementary Figure **8**a plot the EDCs along a momentum cut across Γ (light blue arrow in Supplementary Fig.**8**d). Here we use the data at $T = 75$K, which fulfill the following two conditions: the surviving of quasiparticles and the sufficient intensity of thermally populated spectral weight above E_F. The EDCs in Supplementary Fig.**8**a divided by the Fermi function and symmetrized about E_F are exhibited in Supplementary Figs. **8**b and **8**c, respectively. The effect of Fermi cut-off near E_F is removed after these treatments, and importantly, the dispersion of the spectral peaks (color bars and dashed curves) touches E_F at the Γ point (magenta curves) in both the cases. The results are more clearly demonstrated in Supplementary Fig. **8**f, where the peak positions in Supplementary Figs. **8**b and **8**c are extracted. We find that the plots obtained by the symmetrization and the FD-division techniques (red and blue circles, respectively) almost perfectly matches with each other, both showing the Fermi node state. The consistency between the two different analyses strongly support our conclusion. A further support is presented in Supplementary Fig. **8**e, which plot the the ARPES image after the FD-division treatment. We find the remarkable intensities above E_F (pointed by a tick black arrow), which implies the existence of the conduction band touching on the unoccupied side, as expected in the band calculation.

Supplementary Note 4: Comparison of ARPES data with band calculations: agreement with the TB-mBJ calculation, and disagreement with the GGA calculation

The relevance of band calculation could be judged by directly comparing it with the experimental results. The ARPES is the most direct technique to determine the momentum-resolved band structure, thus it allows us to achieve this aim. Here we carefully compare the ARPES data and two different band calculations of GGA and TB-mBJ, and demonstrate that the TB-mBJ method more correctly reproduces the real band structure in $Pr_2Ir_2O_7$.

There are mainly two differences between the results of the GGA and TB-mBJ calculations as summarized in the left and middle panels in Supplementary Fig. **9**. The first difference is seen in the energy dispersion from Γ toward L point: in GGA, it approaches E_F (red arrow in Supplementary Fig. **9**b), whereas goes toward higher binding energies in the TB-mBJ (red arrow in Supplementary Fig. **9**e). Secondly, the two calculations are distinguished by whether or not the Fermi pocket exists around the L point: the GGA has the doughnut-shaped Fermi pockets surrounding the L point (see Supplementary Fig. **9**a and red dashed circle in **9**b), whereas there is no Fermi crossings in the result of TB-mBJ with fully gapped along the L-W (Supplementary Fig. **9**e).

We summarize our ARPES results in the right-side panels of Supplementary Fig. **9**. We find that the band structure disperses toward higher binding energies from the Γ towards L point in a monotonic fashion (red arrow in Supplementary Fig. **9**h). Secondly, a gap keeps opening all the way from Γ to W point with no indication of Fermi crossings (or Fermi pockets). These two features are reproduced by the TB-mBJ calculation, while disagree with the GGA calculation. The TB-mBJ is thus more relevant than the GGA for the calculation of electronic system in $Pr_2Ir_2O_7$. Most importantly, the absence of pockets

off the Γ point requires the touching of quadratic valence and conduction band just at E_F to hold the charge neutrality, which justifies our conclusion.

Nonetheless, we also find a clear difference between the ARPES data and the TB-mBJ calculation. The ARPES results exhibit a much narrower band width of $\sim$ 40meV in the occupied side than the calculation (>100 meV). Intriguingly, the band width of $Pr_2Ir_2O_7$ is thus more than one order of magnitude narrower than the band structure observed in HgTe [12]. It is actually consistent with the view that the $Pr_2Ir_2O_7$ is a strongly correlated analog of HgTe with a mathematically identical quadratic node at the Fermi energy.

Supplementary Note 5: Dramatic peak suppression at elevated temperatures and aging check after the temperature sweeping

In Fig. 5 of the main text, we investigate the temperature evolution of ARPES spectra measured, and demonstrate a strong suppression of the spectral peak at elevated temperatures. Here we confirm that this is an intrinsic property of $Pr_2Ir_2O_7$ rather than an aging effect on the sample surface.

Supplementary Fig. **10**b and **10**a show EDCs and the same curves symmetrized about E_F over a wide range of temperature from 15K up to 300K. (The same data up to 150K are presented in the main paper.) We used 21.2 eV photons (He discharge lamp), which locate the k point in nearly the midpoint between Γ and L (a green circle in Supplementary Fig. **10**c and a green dashed line in the inset of Supplementary Fig. **10**d). The upturn behavior toward higher energies beyond E_F (arrow in Supplementary Fig. **10**a) is visible regardless of temperature, which is consistent with the theoretical prediction that the quadratic band similar to that in the occupied side is stayed in the unoccupied side (see inset of Supplementary Fig. **10**d). In order to check where or not there is an aging effect on the spectra, we cooled the sample down to the lowest temperature after the temperature scan, and compare the spectral shapes before and after the measurement, as shown in Supplementary Fig. **10**d. The spectral shape just after the sample cleaving is almost perfectly reproduced, which validates that the remarkable variation in spectral shape with temperature is an intrinsic feature of $Pr_2Ir_2O_7$, mostly due to the strong electron correlation.

Supplementary Note 6: Signature of the unoccupied conduction band observed by a laser ARPES with an ultra-high energy resolution

The ARPES is the technique to measure the occupied side of band dispersion. However, the energy states slightly above E_F can be evaluated by examining the thermally populated spectral weight divided by the Fermi function (FD). The electronic system of $Pr_2Ir_2O_7$ is strongly correlated, and the quasiparticle peaks vanish at elevated temperatures. This feature prevents one from a definitive determination of the unoccupied band dispersion with this technique. Nevertheless, we have obtained the upturn behavior in the spectra above E_F (Fig. 5b and Supplementary Fig.**10**a), signifying the existence of the conduction band in the unoccupied side. To verify it further, here we apply the FD-division technique to the data measured by a 7-eV laser ARPES, which is capable of an ultra-high energy resolution ($\sim$2meV), bulk sensitive (a long photoelectron escape depth: $\sim$ 100Å), and high signal/noise ratio measurements. These performances has huge advantages in reproducing the intrinsic spectral shape above E_F by the FD-devision. The k_z position corresponding to 7eV photons is located at the midpoint between Γ and L, where the unoccupied conduction band is predicted to stay at almost the same energy distance from E_F as the occupied valence band (Supplementary Fig.**11**e). Therefore, the signature for the conduction band is expected to be obtained from the spectral tail above E_F at high temperatures.

First, we have tested the FD-division technique to the spectra of the evaporated Au, which is electrically contacted to the samples. We confirmed, in Supplementary Fig.**11**a, that the Au spectra divided by the energy-resolution convoluted FD becomes flat at least up to $3k_BT$ above E_F, meaning that the analysis we use effectively removes the Fermi cut-off effect near E_F. We have accumulated the spectra of $Pr_2Ir_2O_7$ with exactly the same experimental setting as that for the Au measurements, and also applied the identical FD-division analysis to the data in the Supplementary Fig.**11**b. We have reproduced the previous results of spectral peak suppression at elevated temperatures, indicating the significance of strong correlation in this material. Furthermore, the upturn behavior toward higher energies beyond E_F is also obtained in the present data (see the bottom panel of Supplementary Fig.**11**b). Here we emphasize that the thermally

populated spectral weight above E_F seen in the data is large enough to reliably extract the intrinsic electronic state in the unoccupied side by the FD-division technique (see Supplementary Fig.**11**d) at least up to $3k_BT$ above E_F. This feature is exactly expected when the conduction touching-band exists in the unoccupied side. The consistent results from the data sets at different photon energies justify our conclusion.

After the above measurement of temperature scan, we cooled the sample again down to the lowest temperature and compared the spectral shape with that taken just after the sample cleaving. As demonstrated in Supplementary Fig.**11**c, the two curves are almost perfectly overlapped with each other, meaning that the sample aging is negligible. This assures that the strong suppression of spectral peaks at elevated temperatures seen in Supplementary Fig.**11**b is an intrinsic phenomenon for $Pr_2Ir_2O_7$.

Supplementary Note 7: First Principle Calculations for $Pr_2Ir_2O_7$ with Uniaxial Strain

The TB-mBJ paramagnetic band structure calculations show $Pr_2Ir_2O_7$ has a nodal Fermi point, at which the doubly degenerate, quadratically dispersing conduction and valence bands touch at the zone center, right at the Fermi level. This suggests that $Pr_2Ir_2O_7$ is very sensitive to perturbations, such as time reversal symmetry or cubic symmetry breaking terms, giving rise to the possibility of various novel phases. Indeed, the effect of small uniaxial strain near the Γ point can be deduced, up to a sign, by symmetry within the effective mass method [13]. For the proper sign, uniaxial strain is predicted from this approach to open a gap and produce a topological insulator. However, on pure symmetry grounds, it is not clear whether uniaxial compression or expansion is appropriate.

In this supplemental section, we consider this problem microscopically. Uniaxial strain is applied along the $\langle 111 \rangle$ direction, which breaks the cubic symmetry and converts the system into a rhombohedral crystal structure with space group R$\bar{3}$m. Since TB-mBJ has no exchange functional [2], the GGA functional is used for structural optimization, both for the lattice parameters and the atomic positions. In the hexagonal unit cell setting, the in-plane lattice parameter is fixed and the out-of-plane lattice parameter is varied to minimize the total energy. We consider the case of 1% (a =1.01a_0, a_0 = 7.35 Å, the bulk in-plane lattice parameter in the hexagonal cell) and 5% (a =1.05a_0) in-plane tensile strain, which is equivalent to uniaxial compressive pressure along $\langle 111 \rangle$ direction, in Supplementary Figs. **12**c and **12**d, respectively, and 5% (a =0.95a_0) in-plane compressive strain in Supplementary Fig. **12**a. Using the relaxed structure, the TB-mBJ paramagnetic band structure calculation shows that a gap opens with in-plane tensile strain, and the gap increases as the strain intensifies, as shown in Supplementary Figs. **12**c and **12**d. The quadratic band touching state at the Γ point is very sensitive to the cubic symmetry breaking perturbation, and immediately the degeneracy at the Γ point is split as strain is applied. We indeed observe the direct gap at the Γ point is substantial and grows proportionally with strain. However, there is a closing of different states away from Γ, closer to the Z point (π,π,π), which results in a reduced total gap. A small gap, about 4 meV, exists in the case of 1 % tensile strain (Supplementary Fig. **12**c) and the gap is 60 meV in the case of 5 % strain (Supplementary Fig. **12**d). The GGA calculation shows metallic state in the 1 % case and a much reduced gap, 8 meV, with the 5% tensile strain, which is attributed to the well-known fact that GGA and LDA greatly underestimated the gap. On the other hand, by applying in-plane compressive strain (Supplementary Fig. **12**a), $Pr_2Ir_2O_7$ becomes metallic and shows a band crossing along the $\langle 111 \rangle$ direction. This band crossing is indeed expected from the effective mass theory of the perturbed Fermi node, for the strain of sign opposite to that which produces the topological gap [13].

To confirm that the gapped state produced by uniaxial pressure along $\langle 111 \rangle$ direction is topological, we further examine the Z_2 topological invariant. Since the strained structure preserves inversion symmetry, the Z_2 invariant is expressed by [14–16]

$$(-1)^{\nu} = \prod_i \delta_i, \tag{1}$$

where $\delta_i = \prod_{m=1}^{N} \xi_{2m}(\Gamma_i)$ is the parity eigenvalue at the time-reversal invariant momentum i (TRIM), which are tabulated in Supplementary Table 1. The Z_2 class is (1;000), which verifies that $Pr_2Ir_2O_7$ under uniaxial pressure along $\langle 111 \rangle$ direction indeed turns into a strong topological insulator with strong correlelation. Note that the parity eigenvalues at all points except the zone center are essentially already defined in the cubic structure, so that the topological nature is already incipient without strain.

We also consider the surface spectrum of the strained material for further evidence for the topological insulating state. In the latter, an odd number of gapless surface Dirac cones are expected, and we indeed find them, as we now show. To locate the surface state, we first obtain the tight binding Hamiltonian for ⟨111⟩ strained $Pr_2Ir_2O_7$ using maximally localized Wannier functions[17, 18], in the $J_{\text{eff}} = 1/2$ basis. Both the orbital and spinor parts are rotated to the local axes of each inequivalent Ir atom. The rhombohedral unit cell is modified to the hexgonal unit cell, and we consider a thick slab of $Pr_2Ir_2O_7$ to accommodate with the surface state. The slab is fixed to include 73 Ir layers, with identical Ir kagome layers on the upper and lower surface of the slab. Supplementary Fig. **13**a shows the band structure of the bulk and the surface state for $Pr_2Ir_2O_7$ under 5% uniaxial strain on the ⟨111⟩ surface, choosing the large strain to better distinguish bulk and surface states. The Dirac points are located at the $\overline{\text{M}}$ points. On the surface Brillouin zone there are four TRIM, which are located at $\overline{\Gamma}$ and three $\overline{\text{M}}$ points, which are equivalent by 120° rotations. There are three symmetry operations of the crystal with space group R$\bar{3}$m, including the inversion, 3-fold rotation along the ⟨111⟩ direction and two-fold rotation around x direction (orthorgonal to ⟨111⟩). The 2D Brillouin zone also obeys these symmetry operations, making the three $\overline{\text{M}}$ points all equivalent. In total there are three Dirac cones around the TRIM on the 2D surface, which is consistent with the parity argument.

Supplementary References

[1] Perdew, J. P., Burke, K., and Ernzerhof, M. Generalized Gradient Approximation Made Simple. *Phys. Rev. Lett.* **77**, 3865–3868 (1996).

[2] Tran, F. and Blaha, P. Accurate Band Gaps of Semiconductors and Insulators with a Semilocal Exchange-Correlation Potential. *Phys. Rev. Lett.* **102**, 226401 (2009).

[3] Blaha, P., Schwarz, K., Madsen, G. K. H., Kvasnicka, D., and Luitz, J. *WIEN2k, An Augmented Plane Wave Plus Local Orbitals Program for Calculating Crystal Properties.* Vienna University of Technology, Austria, http://www.wien2k.at, (2001).

[4] Millican, J. N. *et al.* Crystal growth and structure of $R_2Ir_2O_7$ (R=Pr, Eu) using molten KF. *Materials Research Bulletin* **42**, 928 – 934 (2007).

[5] Tomiyasu, K. *et al.* Emergence of Magnetic Long-range Order in Frustrated Pyrochlore $Nd_2Ir_2O_7$ with Metal–Insulator Transition. *Journal of the Physical Society of Japan* **81**, 034709 (2012).

[6] Shapiro, M. C. *et al.* Structure and magnetic properties of the pyrochlore iridate $Y_2Ir_2O_7$. *Physical Review B* **85**, 214434 (2012).

[7] Taira, N., Wakeshima, M., and Hinatsu, Y. Magnetic properties of iridium pyrochlores $R_2Ir_2O_7$ (R= Y, Sm, Eu and Lu). *Journal of Physics: Condensed Matter* **13**, 5527–5533 (2001).

[8] Koller, D., Tran, F., and Blaha, P. Merits and limits of the modified Becke-Johnson exchange potential. *Phys. Rev. B* **83**, 195134 (2011).

[9] Feng, W., Xiao, D., Zhang, Y., and Yao, Y. Half-Heusler topological insulators: A first-principles study with the Tran-Blaha modified Becke-Johnson density functional. *Physical Review B* **82**, 235121 (2010).

[10] Ishii, F. *et al.* First-Principles Study on Cubic Pyrochlore Iridates $Y_2Ir_2O_7$ and $Pr_2Ir_2O_7$. *Journal of the Physical Society of Japan* **84**, 073703 (2015).

[11] Laskowski, R., Madsen, G. K. H., Blaha, P., and Schwarz, K. Magnetic structure and electric-field gradients of uranium dioxide: An *ab initio* study. *Phys. Rev. B* **69**, 140408 (2004).

[12] Brüne, C. *et al.* Quantum Hall effect from the topological surface states of strained bulk HgTe. *Phys. Rev. Lett.* **106**, 126803 (2011).

[13] Moon, E.-G., Xu, C., Kim, Y. B., and Balents, L. Non-Fermi-Liquid and Topological States with Strong Spin-Orbit Coupling. *Phys. Rev. Lett.* **111**, 206401 (2013).

[14] Fu, L. and Kane, C. L. Topological insulators with inversion symmetry. *Phys. Rev. B* **76**, 045302 (2007).

[15] Moore, J. E. and Balents, L. Topological invariants of time-reversal-invariant band structures. *Phys. Rev. B* **75**, 121306 (2007).

[16] Hasan, M. Z. and Kane, C. L. Colloquium. *Rev. Mod. Phys.* **82**, 3045–3067 (2010).

[17] Kuneš, J. *et al.* Wien2wannier: From linearized augmented plane waves to maximally localized Wannier functions. *Comp. Phys. Comm.* **181**, 1888–1895 (2010).

[18] Mostofi, A. *et al.* wannier90: A tool for obtaining maximally-localised Wannier functions. *Comp. Phys. Comm.* **178**, 685–699 (2008).
